# Towards a New Model of Verbal Monitoring

**DOI:** 10.5334/joc.81

**Published:** 2020-09-03

**Authors:** Hanna S. Gauvin, Robert J. Hartsuiker

**Affiliations:** 1 Radboud University, Donders Institute for Brain, Cognition and Behaviour, Nijmegen, NL; 2 School of Psychology and Counselling, Queensland University of Technology, Brisbane, QLD, AU; 3 Department of Experimental Psychology, Ghent University, Ghent, BE

**Keywords:** Language production, Auditory word processing, Action and perception, Cognitive Control, Speech perception

## Abstract

As all human activities, verbal communication is fraught with errors. It is estimated that humans produce around 16,000 words per day, but the word that is selected for production is not always correct and neither is the articulation always flawless. However, to facilitate communication, it is important to limit the number of errors. This is accomplished via the verbal monitoring mechanism. A body of research over the last century has uncovered a number of properties of the mechanisms at work during verbal monitoring. Over a dozen routes for verbal monitoring have been postulated. However, to date a complete account of verbal monitoring does not exist. In the current paper we first outline the properties of verbal monitoring that have been empirically demonstrated. This is followed by a discussion of current verbal monitoring models: the perceptual loop theory, conflict monitoring, the hierarchical state feedback control model, and the forward model theory. Each of these models is evaluated given empirical findings and theoretical considerations. We then outline lacunae of current theories, which we address with a proposal for a new model of verbal monitoring for production and perception, based on conflict monitoring models. Additionally, this novel model suggests a mechanism of how a detected error leads to a correction. The error resolution mechanism proposed in our new model is then tested in a computational model. Finally, we outline the advances and predictions of the model.

## Introduction

During speech production we monitor our speech constantly and automatically for errors. As a result, approximately one out of every ten utterances in naturalistic speech undergoes some form of revision (Nakatani & Hirschberg, 1994). Corpus analyses by Meringer (1908), reanalyzed by Nooteboom (1980, 2005a), revealed that 70–80% of phonological errors and 50–63% of lexical errors are corrected. Similarly, when we listen to somebody else speak, we also detect errors. Although the listener cannot influence the production of the error, we still perceive these errors and try to resolve them with the aim of comprehending the speaker. We can detect errors in perception because what we hear is incompatible with our internal knowledge of the world, or common sense knowledge (e.g., a politician says: ‘It will take time to restore chaos and order’), because the speech does not map unto any meaningful message (e.g., ‘I felt so stilly’), or because linguistic criteria are violated, such as grammatical agreement (that same politician: ‘Rarely is the question asked: Is our children learning?’). Especially the latter form of error detection indicates that during speech perception we do not only comprehend (there is no problem in understanding the grammatically incorrect example), but we also monitor for errors. Indeed, without the ability to detect errors during the perception of speech produced by others (other monitoring), it would not have been possible to collect corpora of speech errors, which have been highly influential in shaping our ideas about language production (e.g., Garrett, 1975).^1^ Thus, speech-monitoring is a process that takes place in both speech production (property I) and comprehension (property II).

The most basic and staightforward account of verbal monitoring is that a speaker hears herself speak, and by perceiving her speech she is able to correct errors. Several observations lead to the additional postulation of an *internal* or *inner loop* in addition to the external loop (perception of the speech after production), which allows the producer to monitor the utterance before actual production (property III). A primary observation for postulating the inner loop is that of extremely fast self-corrections. Arguably, the processes of production, interruption, and repair are too fast for monitoring to take place through the external route of perception. If the external route is used for monitoring, the processes of hearing, recognition, and interruption are estimated to take between 350 and 400 ms (Levelt, 1989, Marslen-Wilson and Tyler, 1980, Hartsuiker & Kolk, 2001). Measurements of actual interruptions revealed that error-to-cutoff times are distributed bimodally with two peaks, roughly 500 ms apart with the first peak around 140 ms (Nooteboom & Quené, 2017). Even extremely short error-to-cutoff intervals are observed, in which the erroneous item is cut off almost immediately after initiation (‘v- horizontal’, Levelt, 1989). Clearly, the interruption follows the erroneous production too fast for the interruption to have been processed via production of the phoneme, hearing and processing the phoneme, error detection, and interruption of the incorrect word production, and start of the correct word.

The existence of an internal monitor is further supported by studies demonstrating that participants are able to detect produced errors when external speech is not available, as speech is only produced internally or when speech is masked by a loud noise, in essence forcing participants to use internal monitoring. When participants perform a task only using internal speech (no articulation), they still report the production of errors, demonstrating that indeed internal speech is monitored (Dell & Repka 1992; Oppenheim & Dell, 2008). Sceptical readers are invited here to internally repeat the phrase ‘a quick witted cricket critic’ three times as fast as possible and investigate whether they are able to detect any errors in their internal productions. A number of studies investigated internal monitoring by masking the auditory feedback with noise (Lackner & Tuller, 1979; Postma & Kolk 1992a, b). Noise-masking studies demonstrated that proprioception and bone conductance might be additional available routes for monitoring; under noise-masked conditions errors of place of articulation were detected frequently (84% vs. 92% under normal feedback) but errors of voicing were detected relatively infrequently (19% vs. 72% under normal feedback) (Lackner & Tuller, 1979). However, Postma and Noordanus found no difference in the number of reported errors between silent, mouthed, and noise-masked speech, while more errors were detected in the normal feedback condition. Lackner and Tuller reported that error detection without external feedback is faster compared to normal feedback. This is consistent with the idea that internal monitoring is faster as no articulation and auditory perception need to take place.

A fourth (IV) property of verbal monitoring is that we can exert some control over the monitoring process. When presented with a SLIP task in which certain slips would result in taboo utterances (e.g., TOOL – KITS), participants produced fewer of these slips compared to neutral slip utterances (e.g., TOOL – CARTS) (Motley, Camden, & Baars, 1981, 1982). This indicates that the participant made the SLIP internally, and was able to prevent production with a process of covert editing (Motley et al., 1982). It also suggests that top-down influence can be exerted over the monitoring system. The participant really wants to avoid producing taboo utterances, and is indeed able to intercept and repair the taboo utterance slip quicker than neutral slips. This is further supported by an elevated galvanic skin response that was measured in the taboo trials, even when no slip was made. Similarly a functional Magnetic Resonance Imaging (fMRI) study investigating the neural correlates of inhibition of taboo utterances found increased right inferior frontal gyrus activation on taboo trials compared to neutral trials (Severens, Janssens, Kühn, Brass, & Hartsuiker, 2011), an area of the brain that is thought to play a role in the inhibition of action (Xue, Aaron & Poldrack, 2008).

Further support for context sensitivity comes from the lexical bias effect (LBE). The lexical bias is the tendency for phonological slips to result in an existing word, rather than a non-word (Baars, Motley and MacKay, 1975; Dell, 1986; Dell, 1990; Humphreys, 2002; Costa, Roelstraete & Hartsuiker, 2006; Nooteboom, 2005b). This effect is modulated by context; in a non-word context, the LBE disappears (Baars et al., 1975, Hartsuiker, Corley & Martensen, 2005). Thus, like the taboo word effect, one can consider the LBE as the result of covert editing based on a monitoring criterion that is sensitive to the context.^2^ However, the amount of top-down control that is exerted over the monitoring system seems to be limited: in spontaneous speech (Meringer, 1908) and in experiments with task-relevant speech (Levelt, 1983) the correction rate is similar, while one might expect the participants to want to exert more control in the formal experimental setting compared to spontaneous speech.

Further properties of the verbal monitoring system are revealed by studies with brain-damaged patients. These studies specifically highlight a dissociation in error detection in production and perception. A number of studies have shown patients with a combination of defective self-monitoring during production with intact comprehension, such as patients with neologistic speech (Butterworth and Howard, 1987). Studies with Parkinson’s Disease patients have found impaired monitoring skills and a differential recruitment of monitoring channels compared to healthy controls (Gauvin, Mertens, Marien, Santens, Pickut & Hartsuiker, 2017; McNamara, Obler, Au, Durso & Albert, 1992). In a study of 69 aphasics by Miceli, Gainotti, Caltagirone, and Masullo (1980), no relationship was found between the degree of phonemic output disorder and the number of phonemic discrimination errors. Some of the patients with the most severe output disorder had no discrimination problems. And some patients with a less severe output disorder were incapable of performing the phonemic discrimination in the perception task. Nickels and Howard (1995) examined 15 aphasic patients with phonological production errors, and found no correlation between the proportion of phonological errors in naming and their performance on a series of comprehension tasks. Also a measure of self-monitoring behavior, proportion of attempted error corrections, showed no relation with their performance on auditory comprehension. However, a reanalysis by Roelofs (2005) showed that for phonological processing production and perception skills were correlated.

Marshall et al. (1998) observed subjects who had preserved comprehension, but impaired self-monitoring. Most interestingly, some patients showed successful monitoring of someone else’s speech, despite defective self-monitoring. One particularly interesting case of a dissociation between monitoring in production and perception is described by Marshall, Rappaport, and Garcia-Bunuel (1985). A woman with physically intact hearing suffered from severe auditory agnosia; a near-total loss of the ability to understand speech and non-speech sounds. Despite this loss, she corrected and attempted to correct many of her phonemic errors, while she ignored her semantic errors. These findings suggest that self-monitoring can be performed independently of sound perception (property V).

Marshall et al.’s (1985) case study further suggest that semantic and phonemic monitoring can be lesioned independently (property VI). Relatedly, Oomen, Postma, and Kolk (2005) described a patient with Broca’s aphasia, G., who relied heavily on an internal channel for self-monitoring (when external feedback was masked by white noise, self-monitoring performance remained the same, whereas in the healthy controls self-monitoring decreased). Furthermore G. produced many phonological errors, after which often multiple attempts for repair were made that only resulted in a successful repair 38% of the time. Semantic errors were produced far less frequently, and these were successfully repaired in 64% of the trials. In the perception task, G.’s semantic errors detection was impaired (60% detection, compared to 89% detection by controls), whereas the percentage of phonological errors repaired was similar to controls (84% vs. 86%). So whereas semantic monitoring is impaired in both production and perception, phonological monitoring is only impaired in production. Importantly, this finding is further evidence that monitoring can be impaired separately for semantic and phonological processing. This result suggests that self- and other-monitoring can be performed via different processing routes. Taken together these patient data show that self-monitoring and other-monitoring can be selectively impaired at the semantic and phonological level, and that intact comprehension and intact other-monitoring are not sufficient for correct self-monitoring.

A further property (property VII) of verbal monitoring is that errors are detected, interrupted and a new attempt is made to produce the correct utterance (repaired) (Levelt, 1989). Speech production is monitored for appropriateness, semantic, syntactic, phonological, and prosodic accuracy. The time from error word onset to interruption is referred to as the error-to-cutoff interval. The time from error interruption to production of the repair is referred to as the cutoff-to-repair interval (Blackmer & Mitton, 1991). A number of studies suggest that a speaker interrups an incorrect utterance as soon as possible (Levelt, 1989; Bredart, 1991). Consistent with this, error-to-cutoff and cutoff-to-repair intervals are often very short, sometimes below 200 ms (Blackmer & Mitton, 1991). A number of disfluencies, such as repetitions, (filled) pauses and prolongations are interpreted as a sign that an error is detected before production and are therefore referred to as indications of covert repairs^3^ (Levelt, 1989).

Neuroimaging studies have identified a number of neural correlates of verbal monitoring (propery VIII). Activation of the superior temporal gyrus (STG) is consistently observed in response to speech feedback alterations in fMRI and magneto-encephalography (MEG) studies (McGuire et al., 1996; Hirano et al., 1997; Hashimoto & Sakai 2003; Christoffels et al., 2007, 2011; Tourville et al., 2008; Zheng et al., 2010; Takaso et al., 2010, Shergill et al., 2002). An fMRI study investigating the neural correlates of error detection in speech production and perception revealed a network of areas that was active during error detection for both production and perception (Gauvin, De Baene, Brass & Hartsuiker, 2016). The observed network consisted of pre-supplementary motor area (pre-SMA), dorsal anterior cingulate cortex (ACC), bilateral inferior frontal gyrus (IFG), and anterior insula.

In sum, for a model of verbal monitoring to be complete, the scope needs to include both monitoring of self-produced speech, as well as monitoring of speech produced by someone else. The mechanism needs to be modifiable to context. Furthermore, production and perception need to be independently lesionable, and the same is true for the semantic and phonological level. The model needs to explain how speakers interrupt and correct their errors. A good model needs to be congruent with behavioral and neuroimaging data. A further theoretical consideration is that the theory should be as parsimonious as possible.

In the following section we critically review current theories of verbal monitoring, and evaluate their support given empirical findings and theoretical considerations. This is followed by a proposal for a verbal monitoring mechanism that covers gaps of current theories. Finally, we provide computational evidence for the error resolution mechanism proposed in our new model.

## Overview and critical review of current monitoring theories

### Perception based monitoring: the perceptual loop theory

**Figure 1:**
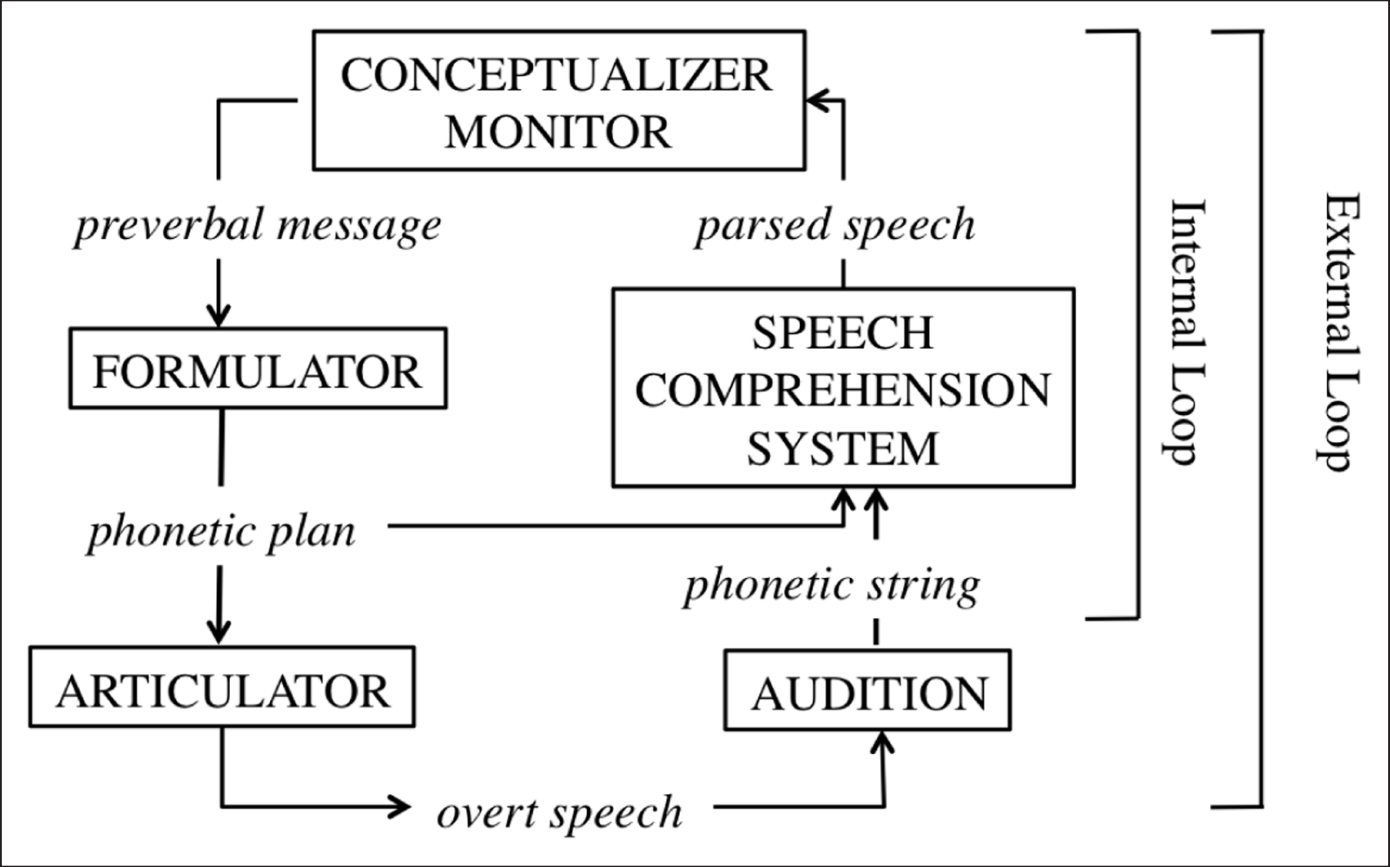
Self-Monitoring according to the Perceptual Loop Model (Levelt, 1983, 1989).

A highly influential and long standing account of speech-monitoring during production is the perceptual loop theory (PLT) (Levelt, 1983, 1989; Indefrey & Levelt, 2004; Indefrey, 2011). This theory assumes that monitoring is dependent on perceptual systems. A schematic overview of the PLT is presented in Figure 1.

#### Architecture of the perceptual loop theory

The PLT assumes that speakers use both external and internal monitoring. The speaker monitors speech by listening to the produced speech (the external route), or via perception of the planned speech before production (the internal route). The external loop thus functions the same for self-produced speech, as for speech produced by others. In the internal loop, a phonemic/phonetic representation is fed into the speech comprehension system (Wheeldon & Levelt, 1995). As a result, the external and internal loop thus both feed into the same verbal monitoring mechanism, and are accomplished identically. The PLT assumes that after detection of an error during speech production, speech is immediately halted and a restart is initiated (Nooteboom, 1980). This principle of halting production upon error detection is known as ‘the main interruption rule’.

A formalized version of the PLT was proposed by Hartsuiker and Kolk (2001). The model predicted the time course of monitoring based on estimates of the duration of production and comprehension processes. This model largely shared the architecture of the PLT but differed in the assumptions about interruption and repair. Specifically, interruptions and repairs were assumed to immediately follow the detection of errors and were planned in parallel.

The neurological substrate for monitoring according to the PLT is the superior temporal gyrus (Indefrey & Levelt, 2004), based on studies demonstrating this area’s involvement in processing feedback alterations. In an updated version of his sketch of the biological basis of language production, Indefrey (2011) additionally suggested that the anterior cingulate cortex (ACC) and supplementary motor area (SMA) are involved in internal monitoring. However, no suggestion was made about the specific role of these areas or why, in contrast to the PLT, internal monitoring would recruit different areas compared to external monitoring.

#### Empirical data

When monitoring, participants detect more errors in the speech of others than in their own speech, however, equal proportions of semantically and form-related errors are detected in one’s own and someone else’s speech (Oomen & Postma, 2001; Oomen & Postma, 2002). This suggests that similar mechanisms underlie error detection during monitoring of self and others’ produced speech.

Evidence in support of similar monitoring for internal (covert) and external (overt) speech comes from experiments showing similar distributions in detecting semantic and phonological errors in overt and covert speech (Dell 1978; Dell & Repka 1992; Postma & Noordanus, 1996). The link between internal and external verbal monitoring was further supported by a study showing speech perception effects, more specifically a uniqueness-point effect, during phoneme-monitoring in production (Özdemir, Roelofs, and Levelt, 2007). The authors argued that this task taps into internal speech monitoring, and interpreted the results as showing that the internal loop is indeed perceived in an identical manner as external speech. However, in this task inner speech is only tested in the absence of external speech (e.g., in silent phoneme monitoring). In a series of experiments in which perception-specific effects (i.e., speech-driven eye-movements) in inner speech were tested in the presence of external speech, no inner speech effects were observed (Huettig & Hartsuiker 2010; Gauvin, Hartsuiker & Huettig, 2013). Furthermore, the uniqueness-point effect observed by Özdemir et al. (2007) is a result of sequential processing that is sensitive to the predictability of the successive segement. It is thus not nescessarily a speech perception effect.^4^

The PLT assumes one (perceptual) monitoring mechanism. However, there is ample evidence for a dissociation between error detection in language production and perception from patient studies, of which a number were listed above. This is irreconcilable with the PLT, as internal self-monitoring, external self-monitoring, and comprehension are all performed by the comprehension system by feeding a stream of perceived speech to the conceptual level, thereby assuming a tight link between error monitoring at the different stages (e.g., semantics and phonology) and between production and perception.

In a series of experiments Nooteboom and Quené (2013, 2017) investigated the relation between the perceptibility of errors in production and perception. In a SLIP production task (2017) the error-to-cutoff times of repaired errors showed a bimodal distribution, with the two peaks roughly 500 ms apart. This fits well with the internal and external monitoring mechanisms proposed by the PLT; presumably early detected errors are detected via the internal monitoring loop and late detected errors via the external monitoring loop. However, in the SLIP production task (Nooteboom & Quene, 2017) masking the speech with a loud noise did not at all affect late error detection rates, which suggests that the late detected errors are not detected through the external loop.

As in the PLT error detection is dependent on the perception of errors, a number of predictions can be derived about an experimental situation in which listeners are instructed to identify consonants from auditory fragments excerpted from SLIP-task data (Nooteboom & Quené, 2013). Specifically, these authors compared reaction times and misidentification rates for initial phonemes of correct production (e.g., *good beer*), undetected errors (e.g., *bood geer*), early detected errors (e.g., *boo…good beer*), and late-detected errors (e.g., *bood gee…good beer*). The authors reasoned that errors, in contrast to correct responses, would often have perceptual traces of both the correct /g/ and incorrect /b/ segment (articulatory blending) and would therefore be perceptually unclear. On the further assumption that the less perceptually clear a segment is, the longer it takes to identify it, the authors predicted that: (1) correct segments can be detected faster than errors; (2) detected errors can be responded to faster than undetected errors; (3) early detected errors can be detected faster than late detected errors. However, only the third prediction was borne out by the data. In contrast to the predictions derived from the PLT, reaction times for errors and corrects did not differ and early-detected errors were even responded to faster than corrects. Additionally, and also in contrast to predictions, late-detected errors were responded to more slowly than undetected errors.

Support for the PLT’s assumptions about interruption of speech production and restart is based on the observation that interruptions do not follow word boundaries but seem to be instantiated immediately after error detection (an exception to this observation are so called *appropriateness repairs* (e.g., ‘a glass’ followed by the repair ‘a tall glass’), which are often delayed until the end of a word (Levelt, 1983). Further support for the main interruption rule comes from a study by Brédart (1991) showing that short words are more often completed before interruption than long words. However, more recent work by Hartsuiker, Catchpole, De Jong, and Pickering (2008) reported evidence suggesting that the interruption is sometimes postponed until the repair is planned (also see Seyfeddinipur, Kita, & Indefrey, 2008). A computational test of the theory was performed by Hartsuiker and Kolk (2001) with simulations. Hartsuiker and Kolk tested whether the observed short error-to-cutoff and cutoff-to-repair intervals were possible in a model using the perceptual loop for monitoring. These simulations showed that error correction via perception is fast enough to explain the short error-to-cutoff intervals, but only with a working inner loop. Importantly, when the inner loop in the model was lesioned, the error-to-cutoff intervals were much longer than in the empirical data. Additionally, the computational model was able to simulate the effect of speech rate on error-to-cutoff and cutoff-to-repair intervals.

More recently, Nooteboom and Quené (2019) proposed an alternative account, according to which at lexical selection multiple candidates are highly active, and during error repair the competition is sustained. As a result, right after error production the correct word is highly active and can be produced right away as a repair. On some occasions, however, the speaker might hold off the repair for strategic reasons when the repair is not readily available.

In a study with altered auditory feedback, Lind et al. (2014a, b) manipulated the auditory feedback of participants performing a Stroop task, such that upon producing ‘green’ the participants hear ‘grey’ in their headphones. Utterances with altered feedback were sometimes accepted as the actual production by the participant. If errors are detected by a comparison of internally generated conceptual and phonological codes with perceived conceptual and phonological codes, one would not expect errors that differ at the conceptual and phonological level to be able to go undetected. However, note that many of the speech exchanges were in fact detected (~ 73% according to Meekings et al., 2015^5^). Furthermore the cognitive load imposed by this variant of the Stroop task might account for reduced error detection, in line with previous studies (Oomen & Postma, 2002). Lind et al.’s results thus do not form a conclusive argument against perceptual monitoring.

The neural activation observed in the STG in response to feedback alterations (e.g., McGuire et al., 1996) has been taken to be evidence of the involvement of the perception system in speech monitoring during production (Indefrey & Levelt, 2004; Indefrey, 2011). However, it is questionable whether neuroimaging studies supporting a role for the STG in verbal monitoring are support for the PLT. Specific about the PLT is that it assumes that *internal* speech is monitored via the perceptual system. The neuroimaging studies cited as support merely point out that altered *external* speech is processed via the perceptual system. The STG has been demonstrated to be active during internal speech production (e.g., Tian & Poeppel, 2010, 2013). However it is not demonstrated that the STG is involved as a function of perceptual monitoring. A more widely accepted assumption is that STG activation is observed as the result of an automatic perceptual prediction following the activation of speech plans. In our view, the observed STG activations are not compelling evidence for the PLT, as it does not clarify anything about the internal monitoring route. Furthermore, an fMRI study investigating internal verbal monitoring during masked production and speech produced by others found no role for the STG in verbal self-monitoring (Gauvin et al., 2016).

#### Theoretical considerations

Several criticisms can be raised against this form of perception-based monitoring. First of all, both the inner and outer loop recruit the perception system so that this system deals with two versions of the same signal with a temporal delay of roughly 500 ms (Nooteboom and Quené, 2017). Nevertheless, speakers do not report the perception of overt speech as an “echo” of inner speech (Vigliocco & Hartsuiker, 2002; Nozari et al., 2011). One theoretical solution would be to assume that one of the channels remains unperceived as a result of selective attention. However, this idea is not supported by data of error detection rates. Error detection rates are frequently reported to be higher in speech with normal auditory feedback, compared to speech with masked feedback (where the participant can only monitor internal speech) and compared to the detection of errors in speech produced by others (where only the external monitoring route can be used), suggesting that in normal speech both the internal and external route are attended at least under some circumstances. However, note that masking auditory input does not always lead to significant changes in error detection (Nooteboom & Quené, 2017; Gauvin et al., 2017).

Second, the PLT leaves the process of comparison rather underspecified. That is, it assumes that the output of the comprehension system, “parsed speech”, is fed back into the system that created the message for production (the “conceptualizer”) and that a comparison takes place at that level. It is unclear, however, what kind of representation of intended speech can be compared with what kind of perceived speech. The fact that we can detect errors at all levels of production (including semantic, phonological, syntactic, and conceptual errors), suggests that the comparison process must be sensitive to errors at all of these levels.

#### Final evaluation of the Perceptual loop theory

The PLT is a highly parsimonious account; the model assumes one system, the perception system, which is necessarily there, by which error detection takes place after production and during comprehension. No system outside the language system is needed to detect language errors. The scope is also excellent; the model explains verbal monitoring in production and perception. However, the empirical data clearly speak against the PLT. The dissociations found between self-, and other-monitoring, and additionally dissociations at the different levels of language processing observed in patients are irreconcilable with the PLT, which assumes one monitoring mechanism for all those components. Data on perception of phonemes in other people’s errors do not support a perceptual monitoring account, while recent findings on the timing of repairs do not support the PLT’s assumption about the coordination of interruption and repair. Finally, the PLT is not supported by neuroimaging data.

### Conflict monitoring

An alternative to a perception-based monitoring system is monitoring via mechanisms internal to the production-system itself (production-based accounts). The earliest production-based account of verbal monitoring assumed that speech is monitored throughout the processing stages, by either several distributed monitors (Laver, 1980), or by a single monitor that inspects the intermediate and output levels (De Smedt & Kempen, 1987; Van Wijk & Kempen, 1987). More recent models of conflict monitoring bear more resemblance to MacKays node structure theory, in which during lexical selection the increased activation of uncommitted nodes leads to an awareness of the erroneous code and subsequent error detection (MacKay, 1987, 1992a, b). No special device is necessary to detect the error. However, it does require the error to become conscious.

#### Architecture of the Conflict Monitoring Account

**Figure 2:**
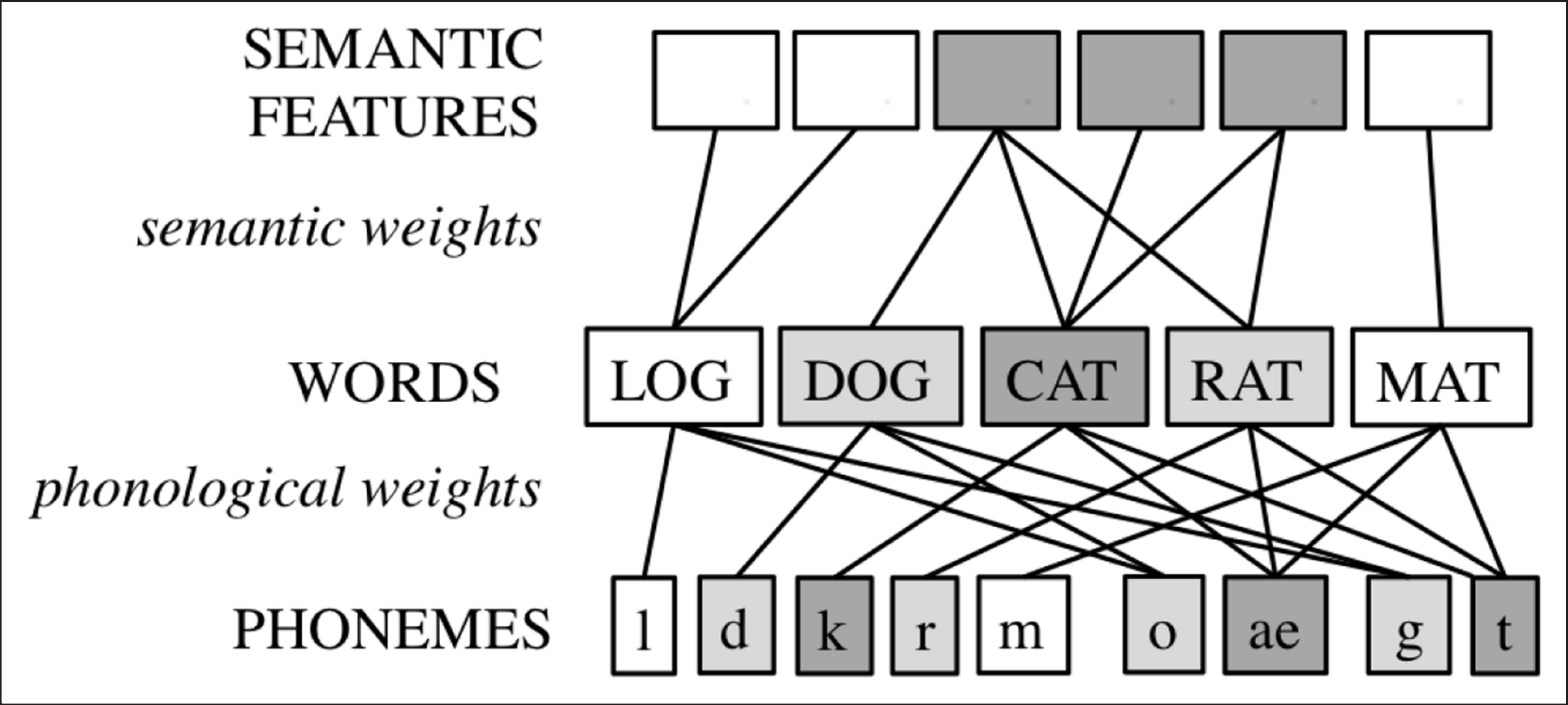
Self-Monitoring according to the Conflict Monitoring Model (Nozari, Dell & Schwartz, 2011).

Neuroimaging work showing ACC involvement in response conflict during speech production initiated the hypothesis of conflict monitoring during speech production (de Zubicaray, 2001). The most elaborate production-based monitoring account is the conflict monitoring account of Nozari et al. (2011). The model builds on domain-general theories of error detection and conflict resolution (e.g., Botvinick et al., 2001; Mattson & Baars 1992; Yeung et al., 2004). The conflict monitoring model proposes that monitoring takes place by determining the conflict between response options in a representational system, where conflict can be seen as a function of the activation levels of units representing these options. In case of a correct production, there is typically only a single highly active unit whereas errors are characterized by multiple units with high activation. The conflict information is then relayed to a domain-general executive center. Nozari and colleagues extended Dell and colleagues’ two-step model of word production (Dell et al., 1997; Foygel & Dell, 2000) with assumptions about conflict monitoring. The model assumes a layer of lexical nodes and a layer of phoneme nodes that are connected via reciprocal connections (see Figure 2). Because of noise in the system and an interplay of different nodes sending and receiving activation, other units than the target one may be highly active (i.e., there is high conflict). Simulations with the model showed that on trials in which the model produced an error, a measure of this conflict was typically much higher than on trials in which the model did not produce an error, suggesting that conflict is diagnostic for the occurrence of an error. As conflict is a layer-specific mechanism, conflict detection can also be layer-specific, and so it is also clear in which layer there is need for conflict resolution. We note that the conflict monitoring model has also been extended to explain how it would function in sentence context (Dell, Oppenheim, and Kitteredge, 2008).

#### Empirical data

The model is supported by computational tests. Simulations demonstrated a strong correlation between patients’ error-detection abilities in a picture naming task and how the model characterized their production skill in terms of lesioning of the semantic and phonological weights (Nozari et al., 2011). The model receives some behavioral support from Nooteboom and Quené’s (2013) study on the perception of errors in the SLIP task. The authors demonstrated that early-detected errors differed more from the late-detected errors than from either undetected errors or correct responses, suggesting that early- and late-detected errors have rather different properties. The authors suggested that early-detected errors – which are perceptually clear – are cases of overly hasty speech initiation (e.g., of /b/) but that activation of the correct segment (e.g., /g/) catches up quickly, leading to high conflict and quick interruption. Late-detected errors on the other hand seem to suffer most from articulatory blending. They might be detected by conflict monitoring or by self-monitoring of overt speech; however, Nooteboom and Quené (2017) showed that late-detected errors did not suffer from auditory feedback masking, ruling out the latter account. Note, however, that the authors leave open the possibility that these late-detected errors are detected by further channels such as proprioception or tactile feedback.

The majority of evidence for domain-general conflict monitoring comes from a growing body of research demonstrating neural correlates that show a high degree of overlap in response to error processing across cognitive processes. Conflict monitoring studies typically show an event-related negativity (ERN or Ne) component in EEG research and ACC/pre-SMA activation in fMRI research during both error production and in high-conflict situations. The Ne component is a response-locked error-related negativity that is observed 50–150 ms after the initiation of an incorrect response in linguistic tasks, with both covert and overt speech responses (Ganushchak & Schiller, 2006, 2008a, b; Masaki et al., 2001; Riès et al., 2011) and in non-linguistic tasks also independent of response modality (Falkenstein et al., 1990, 1991, 1995; Gehring et al., 1993; Holroyd et al., 1998; Vidal et al., 2000). The Ne component is observed independent of the awareness of the error by the participant (Endrass, Franke & Kathmann 2005; Nieuwenhuis et al., 2001; Postma 2000; Ullsperger & von Cramon, 2006). The Ne is also observed in response to situations with high amounts of conflict, such as the Stroop and Eriksen flanker task (for an overview see Botvinick et al., 2001), semantic blocking during picture naming (Ganushchak & Schiller, 2008a), in language decision tasks with homographs (Van Heuven et al., 2008), and in potentially taboo-eliciting trials in a SLIP task (Severens et al., 2011). The amplitude of the Ne is similarly affected by error rate and time pressure across modalities; a low error rate induces a larger Ne after incorrect responses, and time pressure decreases the amplitude (Falkenstein et al., 1996; Gehring et al., 1993; Ganushchak & Schiller, 2006, 2009). Source localization has determined the ACC region as the origin of the Ne component (e.g., Ullsperger, Fischer, Nigbur & Endrass, 2014). Taken together the findings for the Ne component suggest that this is a domain-general response to conflict that arises independently from awareness and comes from a single source that computes a domain-general process. This is very much in line with the predictions of the conflict monitoring account.

The ACC region is broadly connected to motor planning and control systems, and has consistently been observed to be active in neuroimaging research during error production and in high-conflict situations (Coles et al., 1998; Dehaene et al., 1994; Falkenstein et al., 1991; Holroyd et al., 1998; Miltner et al., 2003; Van Veen & Carter 2002; Roger et al., 2010; Debener et al., 2005). The ACC has been shown to be active in a wide variety of tasks, including language, learning and memory, motor control, imagery, and dual task performance (for an overview of experiments, see Botvinick et al., 2001). Most of the studies are consistent with the idea that ACC responds to conflict, and there is broad support for the idea that ACC is involved in cognitive control (D’Esposito et al., 1995; LaBerge 1990; Mesulam 1981; Posner & DiGirolamo 1998). Also in language tasks where participants can freely select from multiple responses there is a consistent report of increased ACC activation compared to repetition or a predetermined response (Andreasen, et al., 1995; Petersen et al., 1988, 1989; Warburton, et al., 1996; Wise et al., 1991; Friston, Frith, Liddle, & Frackowiak, 1993; Frith, Friston, Liddle, & Frackowiak, 1991a; 1991b; Yetkin, et al., 1995; Buckner et al., 1995).

De Zubicaray et al. (2001, 2002) were the first to report ACC involvement during conflict resolution of speech production, and to link this to a domain-general conflict monitoring mechanism. Piai, Roelofs, Acheson, and Takashima (2013) observed the dorsal ACC to be active during incongruent trials across language and non-language tasks, suggesting a domain-general attentional control mechanism. Acheson and Hagoort (2014) investigated whether indeed cross-task correlations of error detection could be found in the EEG signal acquired during three conflict tasks: the Eriksen flanker task, the Stroop task, and a tongue twister task. However, no cross-task correlations with the tongue twister task were found. This led the authors to conclude that the different signatures probably did not arise from a single domain-general conflict monitor. However, as Nozari and Novick (2017) indicate, domain-generality can mean similar computational principles, and does not necessarily entail a shared neural implementation or cross-task resource application. Additionally, it is perhaps not surprising that a highly somatotopically organized structure (Chainay et al., 2004) elicits different signals in response to two tasks that differ on quite a few aspects. There are several important differences between the verbal and non-verbal task that Acheson and Hagoort used that may explain the lack of neural overlap. For instance, the tasks differ in response modality (manual versus vocal), complexity of visual stimulus display, and demands made on working memory (the tongue twister task likely requires memorization). An fMRI experiment by Gauvin et al. (2016) investigating whether overlapping mechanisms were involved in verbal monitoring during production and perception found a network of areas consistently found to be active for error monitoring in the action domain including ACC, pre-SMA, and IFG (Rizzolatti et al., 2001; Wicker et al., 2003; Keysers et al., 2004; Iacoboni, 2005; Botvinick et al., 2005; Shane et al., 2008; de Bruijn et al., 2009; Newman-Norlund et al., 2009; Desmet et al., 2013). These results confirm the predictions of the conflict monitoring account of error detection, and are not directly compatible with a perception-based account.

#### Theoretical considerations

A theoretical issue that can be raised against the conflict monitoring account is that it leaves many aspects of self-monitoring underspecified. Most importantly, it leaves unspecified what happens once the conflict is detected; no theoretical mechanism for repair is proposed. The model in its current state has a principled basis for deciding that an intervention is needed (i.e., in Nozari et al., 2011, a criterion value is reported that would allow for the detection of about half the errors while only falsely raising the alarm for correct productions in less than 1%). However, the model does not specify *how* such an intervention would proceed and *at which stage(s)* of processing. The *how* question concerns the mechanism of repairing: for instance, is a repair a restart from scratch? Is a certain amount of extra activation injected into the system to create the repair? If so, at which level(s)? The *when* question concerns the moment of the intervention relative to conflict detection. Is the repair initiated only after the word has been produced? Or can the repair take place earlier, possibly preventing the error? Arguably, the conflict monitoring model is a model of the control of lexical and phonological selection rather than a model of verbal monitoring; it specifies in great detail how conflict occurs during lexical and phonological selection, but an account of how conflict detection after or during speech production leads to a repair is missing.

#### Final evaluation of the Conflict monitoring model

The conflict monitoring account for verbal monitoring is quite parsimonious as one monitoring mechanism can operate at the different levels of speech production. As the theory is production-specific, error detection during perception is not within its scope. It only deals with conflict monitoring during response selection in a production task. The theory has no account of interruption and repair. The theory is supported by computational and neuroimaging data. Computational simulations of lesions in the model lead to speech production patterns similar to that observed in patients. The vast majority of neuroimaging data available on verbal monitoring are highly consistent with a domain -general cognitive control process during lexical and phonological selection.

### Forward modeling: the hierarchical state feedback control model

Forward modeling accounts of verbal monitoring are based on Wolpert’s proposal from computational neuroscience (e.g., Miall and Wolpert, 1996; Desmurget and Grafton, 2000; Davidson and Wolpert, 2005). Wolpert’s theory was designed to explain movement in motor theory, and considers forward internal models that predict the consequences of actions as a central aspect of motor control and learning. We will discuss two forward model theories of verbal monitoring. In this section we focus on the hierarchical state feedback control (HSFC) model (Hickok, 2012). In the next section, we discuss a further account based on forward models (Pickering and Garrod, 2013a, b, 2014).

#### Architecture of the hierarchical state feedback control model

An important precursor of the HSFC model is the Directions Into Velocities of Articulators (DIVA) model. It is the most detailed and explicit model of speech motor control and uses a feedforward and feedback control architecture to detect and correct overt errors (Guenther, 1994, 1995; Guenther, Ghosh, & Tourville, 2006; Guenther, Hampson, & Johnson, 1998). DIVA is a computational model of motor control during speech acquisition and production. The model is highly neurobiologically specified and supported. Production in this model starts by activating a speech sound map (the auditory target). Speech sound maps project to feedforward articulator velocity maps, that represent the feedforward motor commands for the articulators. This is analogous to Levelts’ proposal of phonetic encoding (e.g., Levelt, Roelofs, & Meyer, 1999; Levelt & Wheeldon, 1994). The speech sound map also projects to auditory and somatosensory target maps. In these forward model projections, the sensory expectations are represented. The auditory and somatosensory target maps send inhibitory inputs to auditory and somatosensory error maps. The error maps receive excitatory activation from auditory and somatosensory state maps. As a result, the activity in the error maps is the difference between the actual and expected sensory states. In case of a discrepancy, an error signal is sent to the feedback control map, which in turn sends a corrective motor command. The model was further expanded to include the assembly and performance of speech sound sequences in the GODIVA model (Bohland, Bullock & Guenther, 2010).

**Figure 3:**
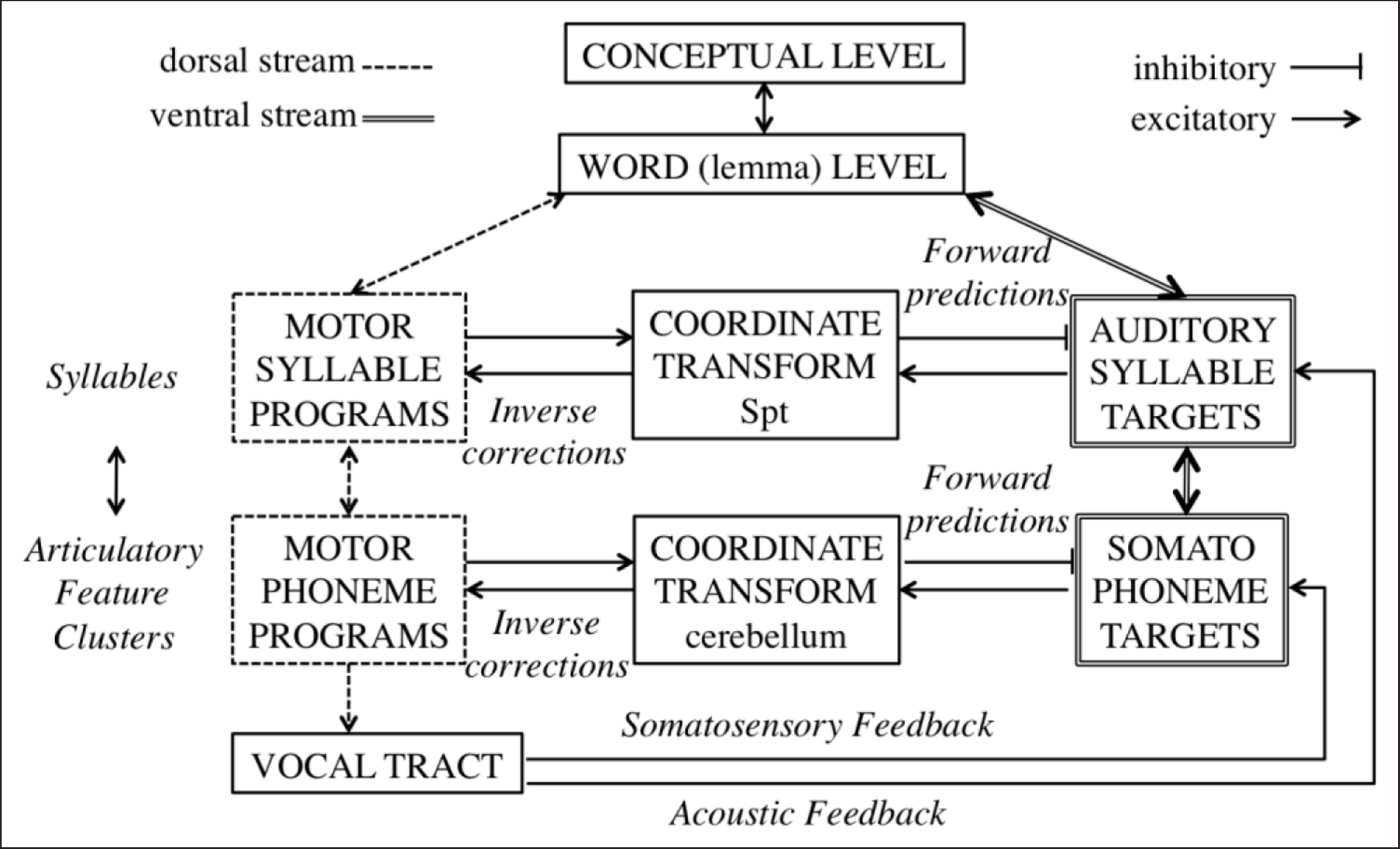
The Hierarchical State Feedback (HSFC) Model of Self-Monitoring.

The HSFC model (see Figure 3) builds further on the DIVA model and expands it with an internal phonemic error monitoring mechanism (Hickok, 2012). The sensory input is processed via the ventral stream, which uses the superior and middle temporal lobe, and processes the signal for comprehension. This stream is an interface between sensory-motor representations. The motor output is processed via the dorsal stream, situated in the posterior planum temporale and posterior frontal lobe, which translates acoustic speech signals into articulatory representations, and forms an interface between auditory and motor representations of speech. These two systems each have their own forward prediction. Furthermore, these two streams are divided into two levels; a higher level that codes speech at a syllable level, and a lower level which codes speech at the phoneme/feature cluster level. A sensory motor translation system is instantiated for both levels; at the lower level the cerebellum mediates between the two processing streams, at the higher level the Sylvian parietal temporal (Spt) area, located within the Sylvian fissure at the parietal-temporal boundary, mediates between the two processing streams.

Activation of an auditory speech form automatically activates the corresponding motor program, regardless of whether there is an intention to speak. The lexical level activates the target of a speech act, and the associated motor phonological representation. To ensure that the activated motor representation will hit the auditory target, the two streams interact. The auditory target then activates the motor representation, which further increases motor activation. The activated motor representation sends an inhibitory signal to the auditory target. When the prediction and the detection match, so no correction is needed, the inhibitory motor-to-sensory efference signal turns off the sensory representation, so that it no longer functions as a correction signal. If an incorrect motor program is selected, the correction signal remains active and will continue to work towards activating the correct motor representation. Internal monitoring takes place in an early phase; errors in motor planning fail to inhibit the correction signal of the sensory representation. External monitoring takes place in a later phase; suppression of the sensory representation enhances the detection of deviation from expectation.

#### Empirical data

The HSFC model is built upon a vast body of neurolinguistic research, which we will discuss only in brief here. One source of evidence is the phenomenon of speaking-induced suppression: neural responses in auditory cortex are dampened when speaking compared to listening to one’s self (e.g., Heinks-Maldonado, Mathalon, Gray, & Ford, 2005). Additional evidence for the HSFC model comes from studies showing efference copy effects (i.e., activation of auditory cortex) during mental imagery. Tian and Poeppel (2010) recorded MEG while participants overtly and covertly produced speech. Around 170 ms after motor estimations by the participants, a response in the auditory cortex was recorded, independent of whether the speech was produced overtly or covertly. In a follow-up study, Tian and Poeppel (2013) demonstrated context-dependent modulations of the auditory cortex to internal simulation. These studies demonstrate a response in the auditory cortex in absence of auditory stimuli, in response to imagined speech production. These studies thus demonstrate auditory cortex involvement in the absence of sounds, which is taken as a role for forward models in speech production. Note that the task instruction was to only imagine articulating – without imagining the auditory consequence. While this seems theoretically plausible, it seems extremely difficult to imagine saying ‘ba’ without the auditory consequence. The instructions to participants was to focus on the movements the articulators go through during this imagined production. Try to imagine this yourself for a moment – like us, you might find it very difficult to successfully suppress the acoustics associated with this imagined production. Potentially the studies thus demonstrate that you cannot disentangle imagining producing from imagining the auditory consequence of producing. Whether this is inherent to the production system or a confound for the task interpretation remains to be tested. Another important note here is that while the HSFC assumes forward models to play a role in articulatory monitoring, it actually assumes that the forward model is suppressive in nature. The predictions measured by Tian and Poeppel were not suppressive but demonstrated the same directionality as auditory cortex responses to actual feedback.^6^ So while they do support the production of prediction in imagined articulation, they do not seem to support the HSFC.

A second body of evidence regarding the role of auditory cortex during speech production comes from fMRI studies with feedback manipulations. Consistently when auditory feedback is altered, activation increases in the auditory cortex proportionally to the manipulation (Christoffels et al., 2007; Tourville et al., 2008; Christoffels et al., 2011; Zheng et al., 2010). However, as pointed out previously, this merely indicates that the auditory cortex is involved in auditory processing, and does not provide robust evidence for a role in monitoring.

The claim that sublexical units are represented separately in the auditory and motor cortex, and the conversion of auditory targets to motor commands was tested computationally with the SLAM (Semantic, Lexical, Auditory, Motor) model (Walker & Hickok, 2016). The model performed well in simulating aphasic patient data. Especially noteworthy is that the model performed well on simulating conduction aphasic naming patterns. Conduction aphasic patients have fluent production, intact auditory comprehension, and good error detection. However, they produce many phonemic errors and have trouble with non-word repetition (e.g., Goodglass, 1992). This pattern is assumed to arise from damage to the link between auditory and motor systems (Anderson et al., 1999; Geschwind, 1965; Hickok, 2012; Hickok et al., 2000).

Finally, TMS stimulation to the right cerebellum leads to longer RTs and an increased production of phonological errors, supporting the view that internal models generated by the cerebellum play a crucial role in phonological encoding (Runnqvist et al., 2016).

#### Theoretical considerations

The HSFC is in essence a domain-general monitoring theory, as the computational principles of feedforward and feedback models and their function in error monitoring proposed have been demonstrated in the motor control domain (Wolpert, 1997; Wolpert, Ghahramani & Jordan, 1995; Kawato 1999; Shadmehr, Smith & Krakauer, 2010). However, a main theoretical concern is that the HSFC’s scope is extremely limited for a language model, as it only deals with phonological and motor processes. On the other hand, the model accounts for this restricted part of the process in a very elegant way, which is strongly supported by brain imaging.

As the scope of the theory is restricted to phonological processing, it is unclear whether and how an extended version of the model could apply to the earlier processing stages of speech production. Especially for the selection of grammatical structure or semantic items it is difficult to imagine how the model would apply, as no sensory feedback is available. One possibility is that monitoring at these levels operates independently. Such an independence between the semantic and phonological processing levels is in line with the patient data above.

Second, the model only handles speech production but offers no account for monitoring perception. One possibility would be that during perception a prediction is made of the upcoming words, as proposed in the forward model theory by Pickering and Garrod (2013a, b, 2014) to which we will turn shortly. Indeed, a suggestion is made to the application in perception: “It [*i.e., the inhibitory input to sensory systems, G & H*] provides a mechanism for explaining the influence of the motor system on the perception of others’ speech” (p. 8 of Hickok, 2012). However, if motor representations were activated in speech perception, the model would be subject to the same criticisms that Hickok himself has on the forward model account of Pickering and Garrod (Hickok, 2013): sensitivity would be decreased for the perception of someone else’s speech. Hickok (2014) rejected this proposal, as the idea behind forward models in production is that deviations can be used to modify the motor plans for production. It is unclear why or how the motor plan would map onto a semantic representation. Instead, Hickok proposed that a ventral stream is involved in perceptual monitoring, independent of the dorsal stream as proposed in the HSFC model.

Third, similar to the conflict monitoring model, the HSFC stops with error detection. There is no account of interruption, repair, and their coordination.

#### Final evaluation of the Hierarchical State feedback control model

The HSFC model provides a very elegant and neurally plausible description of verbal monitoring at the articulatory level. However, it is unclear whether the model is relevant for self-monitoring of human communication more broadly, which requires an explanation of the detection, interruption, and repair of errors at many levels of language production and perception.

### Forward model theory

A hybrid model for monitoring, also inspired by Wolpert’s forward models, is the forward modeling account that has been proposed by Pickering and Garrod (2013a, b, 2014). The model is one of the few that covers both production and perceptual verbal monitoring.

#### Architecture of the Forward Model Theory

**Figure 4:**
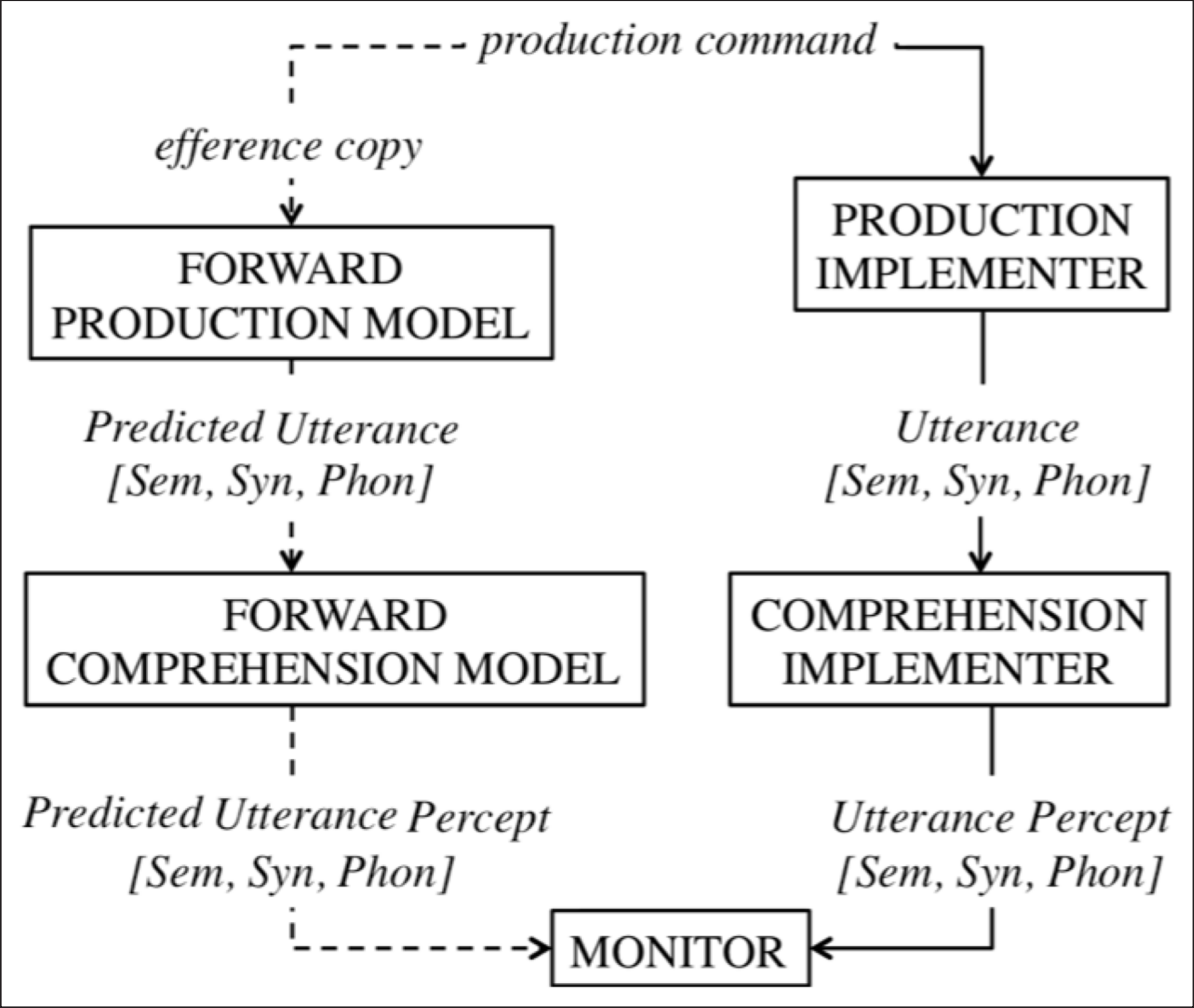
The Forward Model account of Self-Monitoring. Sem is the semantic representation, Syn is the syntactic representation, and Phon is the phonological representation.

In Pickering and Garrod’s forward modeling theory of language production (see Figure 4), a “prediction of the production” is created at each step of the production process, at the semantic, lexical, and phonological level, based on one’s intentions and production outcomes in the past. Each utterance starts with an action command. From this command two processing streams start. The first goes through an action implementer to create a speech act. Next, this act goes through a perception implementer to create a percept. The second stream goes through a forward action model to create a predicted act. This predicted act goes through a forward perceptual model to create a predicted percept. The percept and predicted percept are then compared in a comparator. Comparison takes place sequentially for each level of language production as soon as the percept and predicted percept are created; semantic representations are therefore compared earlier than phonological ones. This is thus different from the PLT where only the penultimate outcome of the production process, namely the phonetic code, is sent to the perception system to compare with the outcome (external speech). Small differences between the percept and predicted percept could be resolved by updating the prediction, whereas big differences between predicted and actual utterance percept, would require an adjustment of the production. Importantly, this mechanism is similarly applied to speech produced by others. The listener uses prediction-by-simulation to predict upcoming words via their speech production system. Similar to speech production the predicted utterance percept, created internally by the listener, is compared to the actual utterance percept. Any deviations will lead to an updating of the prediction of the upcoming utterance.

#### Empirical data

There is abundant evidence that prediction plays a central role in language processing. For instance, Altmann and Kamide investigated anticipatory eye-movements during sentence perception (Altmann & Kamide 1999; Kamide, Altmann & Haywood, 2003). When presented with a visual display, eye-movements are directed towards the picture describing a predicted sentence ending. For instance the sentence fragment ‘The man wanted to ride’ elicited eye-movements towards the picture of a bike, whereas ‘The girl wanted to ride’ elicited eye-movements towards a picture of a carousel. This suggests people create predictions at the conceptual/semantic level. Van Berkum et al. (2005) reported EEG evidence showing that Dutch listeners respond to the grammatical gender-incongruency of an adjective with a noun they expect in the context. This suggests predictions are formed at the morpho-syntactic level. In an EEG study, DeLong, Urbach, and Kutas (2005) demonstrated an EEG response to a violation of expectancies at the phonological form level: if the wrong determiner (*a/an*) was encountered for a highly expected noun (e.g., kite vs. airplane), an N400 effect occurred (for an overview of N400 effects to expectancy violations, see Kutas and Federmeier, 2011). However, note that recently a large-scale replication of the DeLong et al. (2005) study by nine labs failed to observe the N400 in response to the article, suggesting that the phonological form of the upcoming word is not pre-activated (Nieuwland et al., 2018). Additionally, an interactive cascading model and a statistical model (e.g., p-chain model by Dell & Chang 2014) could similarly account for the observed data, without the need for a comparator system, making the forward model less parsimonious than other theories.

A second assumption of the forward modeling account is that production plays a role in language comprehension. One piece of evidence comes from Mani and Huettig (2012) who showed that the productive vocabulary size in young children was correlated with prediction skills in sentence perception. Furthermore, studies from brain-damaged individuals (Nozari et al., 2011) and children (Hanley, Cortis, Budd & Nozari, 2016) suggest error-detection abilities are dependent on production abilities.

If the prediction and comparison mechanism are the same in production and perception, the forward model theory suffers the same criticism as the PLT. That is, patient data as discussed above clearly support a dissociation between error detection in production and perception.

#### Theoretical considerations

For the forward model to be functional, it should be both accurate and impoverished, which are two intrinsically conflicting properties. The prediction needs to be accurate and specific such that an error in the utterance percept can be detected after comparison with the predicted uttterance. An impoverished, reduced, prediction is necessary to allow for speedy processing, so that the prediction can precede the actual utterance. But if we assume the forward model prediction to have both speed and accuracy, why would we still need a slow process of implementing an utterance (e.g., Hartsuiker, 2013)? According to the theory, each utterance is produced twice; once as the intended product and once as a prediction (forward model). It is unclear what the advantage is of producing the same utterance twice, especially as one production is an impoverished version of the other (e.g., Bowers, 2013; Strijkers et al., 2013; Meyer & Hagoort 2013).

In the forward model theory, the assumption is that the predicted percept is an impoverished form of the percept. This, however, makes it unclear how aspects of the percept are corrected that are not part of the predicted percept. If the predicted percept, for instance, does not completely specify all the phonological details, perhaps voicing is not specified, than how can an error in voicing be detected? Meyer and Hagoort (2013) specify a range of related issues regarding the forward modeling account. For instance, while the role for prediction in comprehension is quite clear, the question is whether prediction is still a useful tool if the prediction and construction come from the same mechanism.

#### Final evaluation of the Forward Model Theory

The Forward Model Theory is highly parsimonious, as the same mechanism functions during speech production and speech perception. However, the theory is unparsimonious in that each utterance is produced twice; once as the intended product and once as a prediction (forward model). An attractive aspect of the forward model theory is that it implements predictions during perception as a monitoring mechanism. The scope of the model is also excellent, as it encompasses internal monitoring, external monitoring, and other-monitoring. On the other hand, the theory has no account of interruption and repair. A weakness of this model is that it proposes a very tight link between production and perception, thereby making the theory irreconcilable with patient data. Also more evidence is needed to see if there is a role for forward models between the levels of conceptual, lexical and syntactic processing.

### Review summary

From the review above, we can conclude that each model has its own specific strengths and weaknesses. Taken together, two main problems with all current monitoring theories can be specified. The first is that none of the theories can give an adequate explanation of how verbal monitoring is performed during both production and perception. In some theories, perception is out of the scope, as in the conflict monitoring account and the HSFC. Other theories such as the forward model theory and PLT do specifiy monitoring for production and perception, but these theories cannot account for the dissociation between production and perception monitoring that has been found in the patient data. The second main problem of current monitoring theories is that they fail to explain how the detection of an error leads to the production of a correct item. The theories are formulated in such a way as if the detection of an error is sufficient for correct production. Only the PLT gives a detailed account of the interruption of incorrect production, and the consequent repair, but this account is challenged by empirical data (e.g., Nooteboom & Quené, 2017).

From the theories discussed above, the conflict monitoring account is the only one that has a relatively precise account of the mechanism of error detection. This mechanism (a simple function of the activation level of candidate items for selection) can be easily extended to monitoring in perception. In the next section we propose such an extension of the conflict monitoring theory. An implication of this extension to perception is that the model also shares some properties with the PLT (perception can be used for monitoring external speech and someone else’s speech) and especially forward models (perceptual representations would function as forward models). Importantly, we propose a mechanism that uses the conflict to generate the repair. The error detection-to-repair mechanism is tested below in a computational model.

## Towards a New Model of Monitoring

Here we propose a comprehensive model of verbal monitoring that is able to account for error detection in production and perception. We do so by extending the conflict monitoring model of speech production by Nozari et al. (2011) into a monitoring model for speech production and perception. The main issue with previous monitoring models that account for verbal monitoring in production and perception, is that they have made the two processes so dependent, that a separate lesioning of one of the two modalities, would make verbal monitoring in both modalities impossible. However, the two modalities should not be completely independent, as there is plenty of evidence to suggest production and perception interact. Examples are the perceptive processes during production, such as the integration of perceived sounds into our production (Delvaux & Soquet, 2007; Pickering & Garrod, 2004), and phonetic research on speech imitation demonstrating improved comprehension (Adank, Hagoort & Bekkering, 2010). Therefore, we propose to connect verbal monitoring during speech production and perception in a manner that allows for an interaction between production and perception, but without making the two processes dependent on each other.

By extending the conflict monitoring model into perception, the model still does not provide a full account for verbal monitoring; the model addresses how an error is detected, but not how the error is resolved. An interesting solution as to how an error is resolved comes from the cognitive control literature. Verguts and Notebaert (2008, 2009) proposed that the detection of conflict can lead to adaptation via an arousal response in a neuromodulatory system. This response interacts with ongoing (Hebbian) learning and strengthens the active representations, and increases the strength of the connection between the active representations.

In our view, if the conflict monitoring account for speech production, as proposed by Nozari et al. (2011), can be successfully extended to speech perception, with the addition of a neurally inspired conflict resolution mechanism, this would be an important step forward. Furthermore, as it is a computational model, an extension of the model lends itself for direct testing. Below we first discuss the architecture of the model regarding speech production, speech perception, and how these two are linked. Second, we discuss the aftermath of error detection; how conflict can lead to selection and production of the correct item. This is followed by computational simulations of the proposed conflict resolution mechanism.

### Architecture of the Model

**Figure 5:**
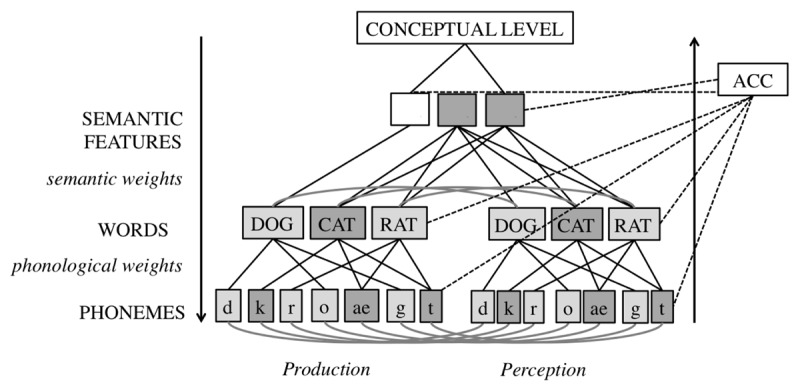
Hierarchical Conflict Model for Self- and Other Monitoring. Speech production and perception have separate words and phonemes, which are tightly connected via links. Arrows indicate the direction of processing.

The architecture we propose (Figure 5) consists of a production network and a perception network. The production network is similar to the model proposed by Nozari et al. (2011); it is an interactive feedback model in which the semantic features in a semantic layer are connected to items in a word layer. The words are connected to phonemes in a phoneme layer. The semantic weight is the strength of the connection between the semantic and the word layer. The phonological weight is the strength of the connection between the word and the phoneme layer. The value of these weights thus determines how strongly the information is transferred between those layers. Speech production happens in two steps. First the semantic features of the target become active. The activation spreads through the network, activating the target word, for instance ‘cat’, but also activating the conceptual competitors at this layer, such as ‘dog’. Via cascading, the activation spreads further down to the phoneme layer. As the model is interactive, the nodes in the lower layer send activation back to the higher layers (feedback). The activation of each node is the sum of activation the node receives from connecting nodes, and this activation is subject to decay and random noise. After n time steps, the highest activated node becomes selected at the lexical level. In the second step an arousal response is sent to the selected lexical node (Nozari et al., 2011; Foygel & Dell, 2000) and activation spreads for another n time steps. Finally, the most active node at each phoneme cluster is selected for the final response (e.g., onset [k], vowel [æ], coda [t]). The amount of conflict is predictive of the occurrence of an error, as demonstrated by simulations (see also the section on simulations below). Further, conflict at each layer is predictive of errors occurring at that layer: for instance conflict at the word layer is predictive of an error in lexical choice but not of a phonological (i.e., nonword) error.

We extend the model with a perception network parallel to the production network, with similar representations in both networks and a tight link between the representations. The assumption that speech production and perception have two distinct systems is supported by the patient data that show dissociations between verbal monitoring in production and perception. The assumption of distinct word representations for production and perception is further supported by research into language acquisition (Gupta & MacWhinney, 1997), dissociations between surface dyslexia and dysgraphia (Weekes & Coltheart, 1996), and dissociations between producing and comprehending nouns and verbs in agrammatism (Kim & Thompson, 2000). Additionally we assume distinct representations at the phoneme level. The phonemic representations at the production level are tightly linked to the motor plans involved in articulation. The phoneme representations at the perceptual level are largely independent of production mechanisms; as a Dutch L1 speaker the first author can perceive the [r] in other speakers of Dutch – in fact this gives her valuable sociolinguistic information about the speaker – but she cannot produce it and uses the [ɻ] instead.

The tight link between the production and the perception system are motivated by the finding of cross-modal priming. For instance properties of perceived sound can be integrated in our speech production (Delvaux & Soquet, 2007; Pickering & Garrod 2004) and (especially semantically related) speech we are trying to ignore can be confused with attended speech during perception (Brungart 2001; Gray & Wedderburn 1960, Lind et al., 2014a, b). Imitating an accent aids in comprehension of accented speech (Adank, Hagoort & Beckering, 2010). Evidence of top-down processes during perception are observed in anticipatory eye movements (Altmann & Kamide 1999; Kamide, Altmann & Haywood, 2003) and expectancy effects in EEG (Kutas & Federmeier, 2011). More evidence that “listening is an active interpretative process, and not a passive reception of an incoming signal” (Cutler, 1982, p. 13) comes from so called hearing errors where the listener misinterprets the heard sentence based on their own expectations (e.g., Garnes & Bond 1975, Bond & Garnes 1980). For example the sentence ‘*Because they can answer inferential questions*’ is perceived as ‘*Because they can answer in French…*’ (taken from Cutler, 1982, pp. 12–13). A very good example of the interpretation process that is at play during perception is an effect known as phoneme restoration. In a study in which a single sound in an utterance was replaced by a burst of noise participants reported hearing a cough simultaneous with the speech, rather than instead of it (Warren, 1970).

The process of perception starts at the phoneme level. Upon hearing speech the relevant phonemes become active, which in turn send activation to the word layer, which in turn sends activations to the semantic layer and so on. The word layer also sends activation back to the phoneme layer.

The production and perception representations are tightly linked, and activation flows automatically via spreading between the production node and the perception node of a representation. If a node in production becomes active, the same node is activated in the perception system, and vice versa. When a word is produced, the representations of the production system are consequently active in the perception system. For instance, if at the word production level the target word ‘dog’ is active, the same node in the parallel word perception layer increases somewhat in activation. In the production system the target word ‘dog’ is selected, and a jolt of activation is sent to the production phoneme layer. Via the interconnections, and spreading from the perception word level, the phonemes in the perception layer also become active. Now the word ‘dog’ is produced, and the perception system is already fully prepared to perceive this word. The activation acts as a prediction of the upcoming percept, and can therefore be used for self-monitoring. In the perception system the phonemes [d] and [o] increase more in activation, and via cascading the word increases in activation as well. After some time the most highly active word is selected, and receives a jolt of activation. This increases the activation at the semantic level, leading to comprehension at the conceptual level.

If an incorrect phoneme were to be selected at the phoneme production level, and an incorrect word would be produced, the perception system would benefit from the spreading activations to recognize the incorrect word. For instance the incorrect word ‘cap’ is produced, instead of the target word ‘cat’. In the perception system the word ‘cat’ would already be active at both the semantic and the word layer.

When listening to someone else speaking, a bottom up process driven by the incoming speech, and a top-down process driven by speech production are started. Pickering and Gambi (2018) proposed a similar theory about the role of prediction during perception. During listening, the perceived words become active first in the perception system, and via the interconnections between modalities the representations in the production system also become active. Based on past experience, the production system activates related nodes, which in turn become active in the perception system, thereby creating a prediction of the upcoming percept. For instance perceiving the utterance ‘I just ate a’ would lead to the activation of edible items in the semantic system. Additionally, experience would contribute to an increase in activation of specific items (e.g., during lunchtime the item ‘sandwich’ would be highly expected). The higher the cloze probability of the word, the higher the activation of the item. In this case, a semantically related word like ‘salad’ will also have high activations. The activation of the items in the production system leads to parallel activations in the perception system, thereby preparing the perception system for these items, thus creating expectancies. When the predicted utterance is met, the active nodes in the perception system increase in activation, until after an amount of time the word is selected for comprehension. When perceived words match the predicted percept, speech perception thus becomes a low-effort process, as the perception of that word is prepared.

The model we propose here has two separate processing streams for production and perception, which can function separately and consequently be lesioned separately. The way conflict is operationalized here, as a function of the activation levels of multiple candidate nodes, means that error detection requires no specific machinery that compares a realized to an intended representation: it rather exploits information that is computed during language production and perception, namely the activation of the nodes in each layer of representations. Subsequent detection of the conflict (and as we will argue below its repair) is done by a domain-general mechanism. This process functions in exactly the same way during production and perception.

In the current model errors can be detected via two mechanisms. The first is via conflict in response selection between highly active nodes in the production layer. The second mechanism is via conflict between highly active nodes in the perception layer. This type of error detection is used in the perception of someone else’s speech, and in one own’s external speech monitoring.

#### Conflict resolution

In the conflict-monitoring model of Nozari et al. (2011), the story ends at the moment conflict is detected. There is no complete account of how the detection of the error is handled. How does the monitor decide which errors to handle? Does an error signal lead to an interruption? Is the interruption followed by a restart? Or is correct selection of a target sufficient?

The question of the aftermath of error detection has been studied, but these studies do not answer all questions stated above. Studies investigating the aftermath of error production have often focused on the temporal coordination between interruption and repair and to what extent strategic components are used, such as postponing the interruption until the repair is planned (Hartsuiker et al., 2008; Seyfeddinipur et al., 2008; Tydgat, Stevens, Hartsuiker, & Pickering, 2011, Gambi, Cop, & Pickering, 2014). Another question concerns the mechanisms of repairing; do you start with a clean slate once you’ve interrupted an incorrect utterance? A number of studies showed that planning of a new word is affected by residual activation of representations pertaining to the abandoned word. Specifically, the experiments showed semantic facilitation and phonological interference effects of abandoned words on repair words (Hartsuiker, Pickering, & De Jong, 2005; Tydgat, Diependaele, Hartsuiker, & Pickering, 2012). A study where participants had to quickly adapt their utterance to make it appropriate for a new context, suggested that utterances can sometimes be repaired by revising the speech plan, rather than plan from scratch (Boland, Hartsuiker, Pickering, & Postma, 2005).

Currently, it is unclear what happens between the detection of an error and the production of the repair. As Botvinick and colleagues put it:

“Existing theories portray the relevant mechanisms as coming into play when the participation is required, but without an account of how the need for intervention is detected or how the intervention itself is triggered. Without a good theory, control remains a sort of homunculus that ‘just knows’ when to intercede. For any theory on cognitive control to be complete, it will need to offer an account of how the system determines when control is required.” (Botvinick et al., 2001, p. 624).

Although the conflict monitoring account does provide an answer to the question ‘when’ intervention is needed (when conflict surpasses a certain threshold), it does not explain how the intervention is performed.

A possible answer to the question of how an intervention is performed might be found in the cognitive control literature. Verguts and Notebaert (2008, 2009) suggested that the detection of conflict can lead to adaptation via an arousal response, which interacts with ongoing learning and strengthens the connection between the active representations. This arousal response works as a non-specific boost or jolt of activation that increases activation of the active representations. A probable candidate for this neuromodulatory boost response is norepinephrine (NA), delivered via the locus coeruleus (LC).^7^ Anatomically the ACC is connected to brainstem neuromodulatory centers, including the LC. Previous research has shown that stimulation of the ACC leads to activation changes in the LC (e.g., Jodoj, Chiang, & Aston-Jones, 1998), demonstrating a tight functional link between these two structures. The LC itself has been demonstrated to play an important role in attention, response selection and task engagement (Aston-Jones, Chiang, & Alexinsky, 1991; Aston-Jones and Cohen 2005), making it an excellent candidate to modulate learning as proposed by Verguts and Notebaert.

Below we integrate the noradrenergic response for conflict resolution for error detection in both production and perception in the conflict monitoring model. Note, however, that the arousal response can in general also be applied to the models above, such as the forward models and PLT, in the following way: on an erroneous trial an error is signaled, by any of the mechanisms described in the models above. The error signal is picked up by the ACC, which sends a signal to the locus coeruleus (LC), which triggers the arousal response, thereby increasing activation of all the active neurons. The top-down arousal boost that is sent into the language processing system causes all active items to increase in activation exponentially, leading to an improved signal to noise ratio. This response strengthens active connections, which are task-relevant, and thereby improves the signal-to-noise ratio, leading to a faster selection of the correct item.

#### Stages of conflict resolution during production

If during the selection process multiple nodes compete for selection, there are two stages at which the conflict can be resolved. The conflict is resolved either before selection of the target, leading to steering (an adjustment during the process leading to the desired outcome), or after selection of the target, leading to error correction. A third function of the model is learning; via the NA response the connection is strengthened.

##### Steering

We assume that if during the selection stage two nodes become highly active, the conflict is detected by a domain-general conflict monitor and a NA response is released. Between the occurrence of the conflict and the resolution via the response some time passes. If conflict is resolved before target selection, the conflict is resolved within the processing level, and resolution is part of the selection process.

##### Correction

If during the selection process a high conflict arises, the highest active node might become selected before the boost response reaches the conflict. As stated above, some time passes between the detection of the conflict and the moment the arousal response reaches the conflict site. In this case a boost of activation leads to a repetition of the selection process. As a result the repair will be selected, in the case of an incorrect target selection, or the correct target will be reselected, if the target already was selected, and sometimes an incorrect ‘repair’ will be selected.

##### Learning

When two items compete for selection, the activation boost increases the chance of correct item selection, and it also increases the strength between the target and its related nodes. Because the connections are strengthened, competition between the competing items will be decreased for subsequent trials in which that target is selected.

### Computational Tests of the Model: Steering and Repairing

To make our theory more concrete we implemented the error detection-to-repair mechanisms as a computational model. Below we first describe the model in some detail and report a baseline simulation that replicates the results of Nozari et al.’s (2011) model. Next, we report simulations with a model that non-specifically boosts the spread of activation after conflict detection (the gain model). We compare the results to alternative models, that specifically boost the activation of the input layer. While these models implement the steering function of conflict monitoring, a further simulation explores the case of later invention, leading to repair.

We first implemented a version of the two stage model of word production developed by Dell and colleagues (Dell et al., 1997; Foygel & Dell, 2000; Nozari et al., 2011). Our initial implementation was based on the Dell et al. (1997) version (the weight-decay model) following the specifications in that paper, as that version provides the basic algorithm on which all newer versions are based. A discrepancy in outcomes, due to a difference in a detail of implementation, was resolved with the help of the first author of that paper. The implementation was then validated by comparing the results to that of an implementation of the model available on the web under a variety of parameter settings.

Next, we created a model with the specific architecture and parameters that Nozari et al. (2011) used. Our implementation of the model contained a feature layer containing 57 feature nodes, a word layer of 6 word nodes (i.e., *cat, dog, hat, mat, fog, log*) and a phoneme layer containing 6 onset nodes (/k/, /d/, /h/, /m/, /f/, and /l/), two vowel nodes (/ae/, /o/), and two coda nodes (/t/ and /g/). Each word node was connected to 10 unique semantic feature nodes with the exception of *dog* and *cat* that had three overlapping feature nodes and 7 unique nodes each. Feature-to-Word connections were bidirectional and each connection has a weight given by parameter *s*. Word nodes were connected to the appropriate nodes for their onsets, vowels, and codas. These connections were also bidirectional; the weight of these connections was given by a parameter *f*.

Processing in the model begins with a boost of activation of 10 units to the features of the target word. Next, during a first step of lexical selection, activation spreads throughout the network for *n* times steps, according to an activation rule by which the new activation is a function of (a) the old activation minus a decay component (parameter *q*); (b) the input, which is the sum of the activation times the weight of all connected nodes (i.e., both forward and feedback); (c) noise, which consists of intrinsic noise (sampled from a normal distribution with mean 0 and standard deviation *SD1*) and activation-based noise (sampled from a normal distribution with mean 0 and a standard deviation of *SD2* times the node’s activation). After the *n*th time step, the word with the highest activation level is selected. Then, the activation level of the winning word is set to 100 and activation spreads for a further *n* time steps. Finally, the phonemes with the highest activation are selected in the onset, vowel, and coda pools respectively. Like Nozari et al. (2011), conflict is determined specifically for each layer, once at the end of lexical selection (but before the jolt of activation to the lexical layer) and once at the end of phoneme selection. Nozari et al. proposed two possible measures of conflict, namely one that was based on the standard deviation of all units in the respective layer and one that was based on the difference in activation between the two nodes with the highest activation. Their simulations showed that the latter measure (i.e., -ln (A_Winner – A_RunnerUp)) was a more sensitive index of errors; hence in our simulations we always used that measure of conflict.^8^

#### Simulation 1: replication of Nozari et al. (2011)

Our first simulation aimed to replicate Nozari et al.’s finding that conflict measured at the lexical level is a sensitive measure of whether the trial will result in a correct production or in a semantic error (by far the most frequent type of error in normal speakers’ picture naming, for which this model was optimized). Like Nozari et al., we used the network version without opportunities for mixed errors (“neighborhood 1”, Dell et al., 2007), and set the parameters as follows: s = 0.04, f = 0.04, q = 0.6, n = 8, SD1 = 0.01, SD2 = 0.16, semantic boost = 10 per feature, lexical jolt = 100. We ran the model for 10,000 trials. The model produced 9,768 correct responses (cat => cat), 216 semantic errors (cat => dog), 4 formal errors (cat => mat; cat => hat), 10 nonword errors (cat => lat), and 2 unrelated errors (cat => log).

**Figure 6:**
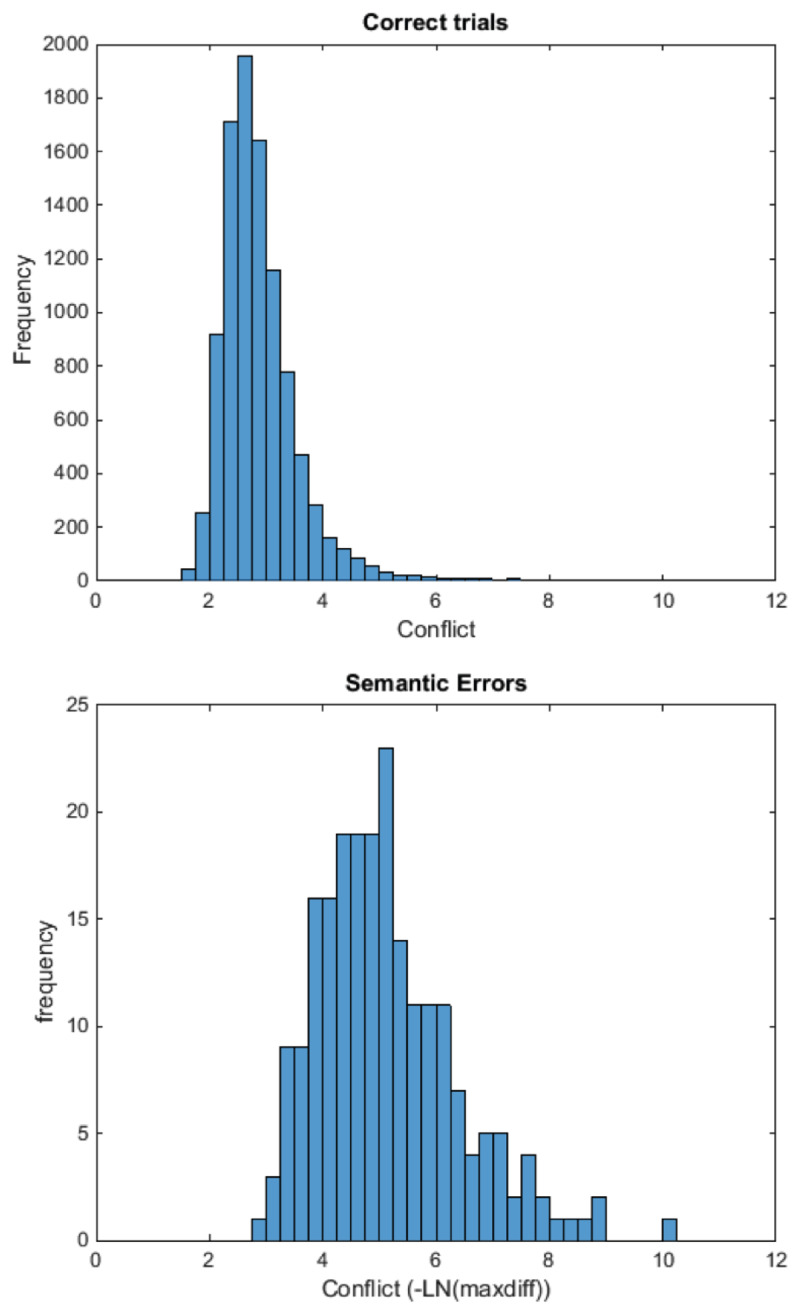
Distributions of conflict at the lexical level (based on the difference between the two nodes with the highest activation) for trials that result in corrects (top panel) and semantic errors (bottom panel). Corrects based on 9,768 trials, semantic errors based on 216 trials.

We determined conflict at the lexical level and determined the distribution of conflict values for trials that resulted in correct responses and in semantic errors (i.e., the final outcome after phoneme selection). Mean conflict for correct trials was 2.88; mean conflict for semantic errors was 4.96. These distributions are shown in Figure 6. As is clear from the figure, conflict allows for good discriminability between correct responses and semantic errors. Indeed, Cohen’s d (a measure of discriminability) was 3.28 which is comparable to the value of 3.26 obtained by Nozari et al. (2011) and indicates good discriminability. Nozari et al. further used a model to estimate a criterion value, given the trade-off between hits (errors detected as such) and false alarms (corrects that are mistakenly classified as errors). That model converged on a hit rate of 47% and a false alarm rate of about 1%. In our simulations, we decided to use two candidate criterion values: a value of 5.25 corresponded to a false alarm rate of 1% and a hit rate of 38%. We also used a value of 4.96, which corresponded to the median conflict value of semantic errors, hence a hit rate of 50%. These values were chosen so that we would have comparable hit and false alarm rates as Nozari et al. (2011).

#### Simulation 2: A conflict-repair model

**Figure 7:**
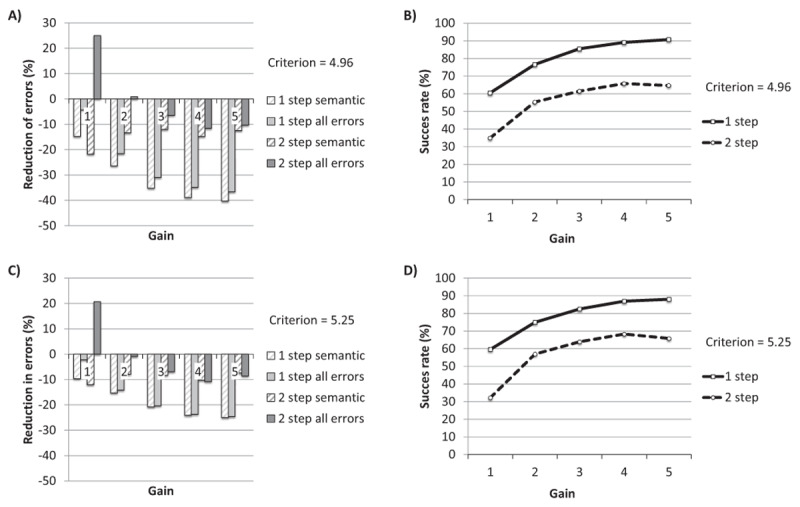
Panels **A** and **C**. Reduction in semantic and all errors for each combination of gain and time steps for criterion values of 4.96 and 5.25 respectively. Negative bars mean that the intervention reduced number of errors. Panels **B** and **D**. Proportions of successfully repaired errors for each parameter combination.

We created a version of the model that responded to high conflict at the lexical level by delaying lexical selection with a small number of time steps (i.e., either 1 or 2 steps) and by temporarily boosting processing. Our hypothesis, based on proposals in the literature on conflict monitoring in cognitive neuroscience (Verguts & Notebaert, 2008, 2009) is that a high conflict signal is a trigger for the release of a neurotransmitter that non-specifically enhances processing. The effect of a neurotransmitter is typically to modulate information processing at the level of the synapse, and indeed Verguts and Notebaert suggested that the release of the neurotransmitter would specifically affect a parameter determining the connection strength (i.e., learning rate). We therefore opted to simulate the processing boost by temporarily adjusting the connection strengths between all nodes. In particular, during a brief interval following conflict detection, connection strengths are multiplied with a gain factor. This gain factor is non-specific (even though conflict detection is layer specific): it affects both downward and upward connections, and it affects not only the connections from features to words but also the connections from words to phonemes. We tested 20 versions of the model (each run for 10,000 trials) crossing two criterion values (4.96 and 5.25), two extra time steps values (1 or 2), and 5 values of the gain parameter (1, 2, 3, 4, or 5). Note that gain = 1 corresponds to the normal model; this shows the baseline effect of giving the models 1 or 2 time steps extra. For each model, we determined error frequencies as usual. We also determined the proportion of repairs that were successful. A successful repair was defined as a repair that resulted in correct selection. Inevitably, this includes cases where lexical selection would also have been correct if there would not have been an intervention (i.e., correct responses that happen to have high conflict); this number is considerable given the high baseline frequency of correct responses. The reduction in number of semantic errors and in all error types combined for each combination of gain and extra time steps is given in panel A of Figure 7 for a conflict criterion of 4.96 and in panel C for a criterion of 5.25. The proportions of successful repairs for both values of the criterion are provided in panels B and D.

The figure suggests that providing one or two time steps extra in a high-conflict situation may by itself (gain is 1) reduce the number of semantic errors slightly. Note however that the reduction in semantic errors in that situation (especially when two time steps extra are given) comes at the expense of an increase in other error types. Most importantly, the figure clearly shows a near-monotonic decrease of semantic errors and all errors combined with an increase in gain. Indeed, the number of successful repairs increases near-monotonically with gain. At the highest gain tested (5), about 90% of repairs result in correct lexical selection. The increase in error reduction is near-linear between gains 1 and 3 and then levels off. Thus, it seems that a transient, non-specific arousal response spreading when there is high conflict can successfully steer lexical selection (to the extent that almost all high conflict trials result in correct productions).

There is also a clear effect of number of time steps: performance is poorer with two steps extra rather than with one step. This is a consequence of the activation dynamics in the network, in which more than half of a node’s activation decays with every time step. This means that activations around selection time are very low in comparison to the noise in the system, thus dampening the positive effect of gain somewhat. The pattern of data is very similar for simulations with a conflict criterion of 5.25 (corresponding to a false alarm rate of 1%) and 4.96 (corresponding to a hit rate of 50%). Of course, the reduction in number of errors is larger for the lower value, as more candidate errors would lead to an intervention. Interestingly, the large reductions in numbers of non-semantic errors were accompanied by small increases in other types of errors when the criterion was 4.96. These increases did not occur to the same extent when the criterion was 5.25.

One way to think of this conflict-based booster mechanism is that it improves the signal-to-noise ratio: thus, a network with this mechanism should yield comparable results to the standard network with a lower than normal noise setting. To compare the effect of boosting to that of a less noisy model, further simulations with the basic model (without boosting) decreased the value of intrinsic noise (*SD1*) until a comparable number of semantic errors or total errors was observed as in the model with the settings: gain = 3, criterion = 4.96, 1 step extra. We found that a comparable reduction in semantic errors (gain model 35.2%, reduced noise model 34.7%) corresponded to a decrease of the intrinsic noise parameter to .007 (noise reduction of 30%). However, the gain model does not reduce the number of non-semantic errors. A comparable reduction in *all* errors (gain model 31.0%, noise reduction model 30.6%) corresponds to a decrease of the intrinsic noise parameter to 0.008 (noise reduction of 20%).

#### Simulation 3: comparison to models with a specific boost

It is important to note that the gain model reduces the number of errors substantially, but that a similar reduction can also be achieved by models that intervene at specific layers or representations rather than providing a more general boost. One such model would specifically boost the activation of the feature nodes (i.e., one might think of this as a loop in which a node detecting conflict at the lexical level sends activation to all nodes at the level of features). Such an arousal response is likely to be effective, because despite strong decay and the effects of noise and (noisy) feedback, the pattern of activation at the feature level is bound to be correlated with the pattern after the initial boost. Boosting this activation will therefore likely steer lexical selection in the correct direction. In our simulations, the network multiplied the activation of every feature unit with *mf* if conflict exceeded the criterion. As before, lexical selection was delayed with 1 or 2 extra time steps. As in the simulations with the gain model, there was a positive effect of the strength of the boost that leveled off at higher levels; performance was better with 1 extra time step than with 2. Performance of the model with mf = 3 were similar to those of the gain model with gain = 3 (Table 1).

**Table 1:** Comparison of different repair models.

	Change in error rate (%)		

	Semantic	Total	Successful repairs (%)

Gain model (gain = 3)	–35.2	–31.0	85.5
Feature layer (mf = 3)	–33.8	–29.7	84.3
Target features (fr = .03)	–37.0	–32.2	86.7

*Note*: Criterion = 4.96, one extra time step. Each model run 10,000 times.

A model that would repair in an even more specific way would simply re-activate the semantic features of the target word; thus, this model would not only boost the activation of the input (feature) layer but would specifically boost the activation of the correct nodes at that layer. To simulate this mechanism, we ran the model and added a fraction of the semantic boost to the features of the target word and delayed selection with one or two time steps as usual. Even small fractions of the original boost resulted in very high successful repair rates. If this fraction was 0.019 or higher, 100% of the repairs resulted in correct productions, but even much lower fractions already resulted in near-ceilings rates of successful repair. If the fraction was set at 0.019 and the criterion at 4.96, the model produced (after repair) 9876 correct responses, 107 semantic errors, 7 formal errors, 10 non-word errors, and 0 unrelated errors. Thus, under optimal circumstances about 50% of semantic errors can be prevented (as is to be expected, given that a criterion value of 4.96 corresponds to median conflict of semantic errors). If the fraction was lowered to .003, model performance was similar to that of our gain = 3 model (Table 1). Such a fraction may seem very low, but it is in proportion to the activation levels of the feature layer, which have decayed strongly during the previous n time steps.

#### Simulation 4: A model of overt repairs

The models described thus far implement the monitoring function of steering the production process: when high conflict suggests that something goes awry, an intervention takes place that puts the model back on track. A detection criterion that leads to a reasonable number of hits (e.g., about 50%) has the disadvantage that there will also be many false alarms (>1%) in light of the very high baseline frequency of correct productions. Arguably, such false alarms do little damage as they invariably lead to correct outcomes (i.e., repairs of corrects do not introduce new errors); their only consequence is a slight delay in processing.

In contrast, false alarms are more problematic in models that respond to conflict later (after the word’s phonemes have been selected) and thus generate overt repairs (*cat… dog* or *k….dog*). In such models, false alarms might lead to word repetitions (*cat cat*) or part-word repetitions (*k…cat*). We created a version of the model that computed conflict at the lexical level but that initiated a repair of high-conflict trials only after phoneme selection. The model repaired by starting afresh (reapplying the jolt of activation to the correct word’s feature nodes). In one version, this jolt was added to the activation levels of the features and activation spread for n + n time steps as usual (corresponding to a model where the repair process can be influenced by residual activation of the earlier trial). In another version, all activation levels were reset to 0 before applying the boost (corresponding to a model that has completely “wiped the slate clean”). We observed, however, that in both models repair success was close to ceiling. In fact, both models produced repairs on 2.49% of the trials. Of these, 1.07% were semantic errors that were corrected and 1.35% were correct responses that were repeated (the remaining .02% were failed repairs of semantic errors and .03% were “repairs” that changed a correct item into an error). There remained 1.11% uncorrected semantic errors. Thus, with these settings about 50% of semantic errors is corrected, but at the expense of introducing 1.35% repetitions.

### Discussion of simulation studies

The simulations with these versions of the conflict monitoring model demonstrated that the neurally inspired repair mechanism we proposed, namely aselective boosting of activation spreading throughout the production network, intercepts and/or corrects errors effectively (equivalently to a reduction of noise in the network of 20%–25%). Such interventions seem particularly useful when they happen early on and so can steer lexical selection back on track (steering function).

## General discussion and conclusion

The model we have described above has several important advantages compared to the existing models discussed at the beginning of the paper. First of all, our model described not only how errors are detected, but also what mechanisms come into play to resolve the conflict. We propose that the repair of an error is mediated by conflict detection by a domain-general monitoring system, located in the ACC, and a subsequent NA boost from the locus coeruleus. This NA boost increases activation of all active items, thereby increasing the signal-to-noise ratio, allowing for a fast (re)selection of the correct item. Additionally, we propose that the NA boost strengthens the connections between the active items (note that we have not yet implemented this learning mechanism).

A second strength of the model above is that we specify for both speech production and speech perception how error detection and conflict resolution take place. In both processes, conflict arises as two or more items compete for selection. This conflict is resolved in both modalities by a domain-general conflict monitor as described above.

A third strength of the model is that it proposes a tight link between production and perception, without reduplicating the production process. By assuming a cascade of activation via tight links between the nodes in the production system and the perception system, the production system can be involved in predicting upcoming utterances. And the perception system can use the cascading information from the production system for verbal self-monitoring via the external loop. It also gives a natural explanation of how perceived sounds can be integrated in our production.

A fourth strength of the model is that this model accounts for the patient data discussed above. Although production and perception are connected via tight links, neither module (intact production or perception) is a prerequisite for monitoring in the other module. Monitoring during production and perception can take place independently, and can be lesioned separately.

### Advances and predictions of the model

In sum, we propose a hierarchical feedback model with conflict resolution by a domain-general monitor. Conflict arises as multiple nodes increase in activation, thereby competing for selection. This conflict is picked up by the domain-general conflict monitor in the ACC, and consequently resolved by an NA response that boosts all active nodes. As a result of the boost, the signal-to-noise ratio of competing items increases and activations are strengthened.

This model is able to explain how patients with faulty self-monitoring exhibit intact other-monitoring, unlike the PLT or forward model theory. It also explains how intact self-monitoring can occur without using the external route for self-monitoring, as suggested by the patient data discussed above. If the connections between the production and perception representations are lesioned, the person can still exhibit intact self-monitoring and intact other-monitoring, but is impaired in predicting upcoming speech.

#### The effect of context on monitoring

Due to the architecture of the model, in which production and perception can interact, our model makes specific predictions about the effect of context on the monitoring process.

##### Unconnected speech

In unconnected speech, such as the perception of a single word in an experimental setting, conflict would only arise if the perceived word contains enough correct phonemes to activate a word at the semantic layer via the reciprocal connections. Upon perceiving ‘cactut’, ‘cactu’ leads to activation of the lexical item of ‘cactus’, which leads to competition between the expected ‘s’ and the perceived ‘t’. Upon perceiving ‘cap’, where ‘cat’ is intended, no error is detected, as ‘cap’ is a valid entry that does not lead to competition.

##### Connected speech

In connected speech, or speech within a context, the production system comes into play. When the predicted utterance is wrong, the incoming speech will activate different nodes than those selected by the production system. The subsequent competition is resolved by the ACC – LC system. For instance, you hear the utterance ‘Last night before I went to bed, I was brushing …’. While listening, you predict that the upcoming words will probably be ‘my teeth’ or ‘my hair’. In fact, the sentence ends with ‘my teef’. In this case the nodes belonging to ‘my’ are highly active, the node is selected, and the activation decays. ‘teef’ activates the phoneme units ‘t’ ‘e’ and ‘f’. As the production system has already activated the semantics and phonology of ‘teeth’, the phoneme units ‘t’ and ‘e’ will increase the activation of the semantic node. Competition between the ‘th’ and ‘f’ is resolved by the ACC – LC system, selecting the already highly active node ‘teeth’.

#### NA and conflict resolution

As the current account makes a direct link between NA release and conflict resolution, a clear prediction is that conflict leads to an NA release, with the amount of NA release related to the amount of conflict. Furthermore, as the NA release leads to a strengthening of the connections, we also have predictions with respect to the aftermath of conflict resolution.

##### Pupil dilation

A tight link exists between the neurotransmitter NA and pupil dilation (Rajkowski et al., 1994; Phillips et al., 2000; Samuels & Szabadi 2008; Sterpenich et al., 2006). Under constant illumination the NA levels are reflected in the dilation of the pupil. If indeed NA is responsible for resolving conflict in linguistic processing, then the amount of conflict should be reflected in the dilation of the pupil. In both visual and auditory ambiguity resolution an increase in pupil diameter is measured just before a perceptual switch was reported (Einhäuser, Stout, Koch & Carter, 2008). The magnitude of the observed dilation was indicative of the subsequent duration of perceptual stability. Pupil dilation has also been measured during discourse processing, and in this study it was found that correct prosodic cues were indeed related to the smallest dilations compared to uninformative prosodic cues (Zellin, 2011). Similarly Zekveld et al. (2014) found pupil responses to different degrees of audibility of speech. When listening to someone speak, largest dilations were observed when the participant heard someone else speaking simultaneously. Smaller dilations were observed when the speech was masked by random noise. And the smallest dilations were observed for noise-vocoded speech. Pupil dilation has also been reported to be a reflection of word retrieval effort in bilinguals (Schmidtke, 2014); low word frequency and high neighborhood density were related to high pupil dilation, as predicted on the basis of our model. High proficient bilinguals showed, in comparison to low proficient bilinguals, an earlier pupil response and a smaller effect of neighborhood density and frequency. These findings are very much in line with our model, in which an NA response is released for conflict resolution.

##### Drugs

Alpha-blockers inhibit the firing of cells in the LC, thereby reducing the release of NA. Alpha-blockers are used for the treatment of anxiety, panic disorders, and PTSD. A direct effect of Alpha-blockers on self-monitoring is expected, as conflict resolution will be heavily impaired. The production of errors will increase and fewer corrections will be made.

##### Neurological disorders

Two patient groups typically associated with abnormal NA functioning are schizophrenic patients and patients with Alzheimer’s disease. In schizophrenic patients increased NA levels are measured in the cerebro spinal fluid (CSF) compared to age-matched controls (Kemali et al., 1982; Lake et al., 1980). Treatment of these patients with clondine or guanfacine (α_2_ adrenergic agonist) causes reduced functioning of NA receptors, improved cognitive functioning as measured by learning, delayed recall, and the Trail B task (Fields et al., 1988; Friedman et al., 1999). Deficits in self-monitoring have been hypothesized to be the cause of the auditory illusions in some schizophrenic patents; what exactly the effects are of abnormally high NA levels remains to be investigated (e.g., Wilkinson, 2014; Hommes et al., 2012).

Alzheimer’s disease is associated with a loss of up to 70% of NA projecting cells in the LC (Heneka et al., 2010). However, Alzheimer’s disease also leads to loss of neurons and synapses in the cortex and sub-cortical regions, including the frontal lobe, parietal lobe, temporal lobe and cingulate gyrus (Wenk, 2003), thereby making this disease a less ideal candidate to investigate the role of NA in language processing.

##### Aftermath of conflict resolution

During conflict resolution the arousal response increases the strength between the connections according to Verguts and Notebaert (2008, 2009). This is a form of learning. As the arousal response is in function of the amount of conflict, learning is also in function of the amount of conflict. So once conflict is resolved, a subsequent encounter of the situation should not lead to as much conflict. If, for instance, conflict arises between ‘tea’ and ‘coffee’ at a lexical level, the arousal response will strengthen the connections between the semantic and lexical level representations of ‘tea’.

As the arousal response of NA strengthens the connections between the active semantic and lexical nodes, conflict between nodes in the trial increases the conflict of the active items the next time they need to become active. So conflict resolution between two semantically related items (‘dog’ and ‘cat’) leads to increased interference in the production of these items in following trials.

If on trial n ‘chest’ and ‘brest’ compete, the production of ‘torso’ in trial n + 1 is subject to interference as the semantically related item ‘chest’ was strengthened in the previous trial. This latest prediction is confirmed by the finding of cumulative semantic interference (e.g., Brown, 1981; Costa, Strijkers, Martin, & Thierry, 2009; Howard et al., 2006; Navarrete et al., 2010; Oppenheim et al., 2010; Runnqvist, Strijkers, Alario, & Costa, 2012): naming latencies for items of the same semantic category increase at each consecutive trial.

The hierarchy of the model predicts that during production, a conflict at the semantic level with a late conflict resolution will also lead to a conflict at the phonemic level. Thus, shortly before the misselection of ‘dog’ for ‘cat’, the lexical items ‘dog’ and ‘cat’ will both cascade activation to phonological coding systems. This leads to high conflict at the phonological level. A conflict at the phonemic level will, however, not lead to conflict at the semantic level. The feedback activation, here from the phonological level to the semantic level, is not strong enough to create competition at a higher level. As a result competition at the phonemic level does not strengthen the connection of competing semantically related items, but competition at the semantic level does strengthen the connection at the phonological level of phonologically related items. So competition between ‘cat’ and ‘dog’ should facilitate the production of ‘log’ in the subsequent trial, but competition between ‘dog’ and ‘log’ should not facilitate the production of ‘cat’ in a subsequent trial.

As the model is a direct adaptation of the conflict model of Nozari et al. (2011), semantics and phonology can be lesioned separately. The strength of the links between the different layers can be weak or strong, independent of the strength of connections to other layers. This is also a prerequisite to account for the patient data discussed above. Similar to the model of Nozari et al. (2011), the current model can be lesioned by decreasing the strength of the connections. In the case of weak connections at a specific layer, the boost of activation will be of relatively little help; when the connections are weak, only few errors will lead to a conflict high enough to trigger the boost response. And the boost response will often not lead to a correct target selection. So people with weak phonological processing will produce a lot of phonological errors, which most often remain undetected. When an error is detected, the repair will often be incorrect.

##### Proficiency

If monitoring of others’ speech is aided by interactions with the production process, then the level of monitoring success of the speech of others will be correlated with the production skills. Note that this is testable in both children and L2 learners.

### Parallels with forward models

In the current model we consider cascading and feedback from lower levels as a form of forward modeling, as also suggested by Dell (2013). In forward model theories, the forward model is an impoverished version of the production command. In a hierarchical feedback model the activation cascading down also increases the activation of connected nodes in the next layer, but to a much lesser extent than if the nodes were committed parts of a representation. The goal of the forward model is to ease the selection process, thereby speeding it up. The cascading of activation fulfills the same function. By already increasing activation of the connected nodes, the construction of representations at those levels is prepared. And finally, the feedback from the lower layers to the higher layers in a cascading model allows information of the lower layers to become available to the higher layers, so these different layers can be coordinated, much like the forward model does for the production command. A further parallel with forward models is that during production, activation spreads to the perception system. This can thus be considered a ‘forward model’ that can be used for monitoring the external percept. However, note that this interpretation of an intergrated forward model differs quite from the proposals put forward by Hickok and Pickering and Garrod discussed above which constitute an auxiliary dedicated prediction mechanism (Pickering & Clark, 2014).

In this paper we proposed a conflict monitoring model for error detection in production and perception. We acknowledge the role of the production system in the perception of language via reciprocal connections through which activation cascades. As a result, perceptual representations become active during production, allowing for external self-monitoring. And during perception, units become active in the production system, whereby prediction of the upcoming percept can be made. Additionally we propose a mechanism for verbal monitoring via a domain-general conflict detection mechanism, and a connected mechanism that resolves the conflict via a non-specific activation boost. Computational simulations confirmed that the proposed boosting mechanism can successfully steer and correct target selection, validating the possibility of a domain-specific conflict monitoring mechanism for verbal monitoring.

## Footnotes

However, see Cutler (1982) and Meyer (1992) for overviews of limitations on speech error detectability and its effect on speech error corpora.

Note that the LBE can be viewed as a detection bias, where it would be easier to detect a non-word as erroneous, but also as a correction bias, where some detected errors are corrected more often than others; however, there is no evidence that supports the latter theory (see Hartsuiker, 2006).

Alternatively the repetitions and pauses have been interpreted to result from a heavy processing load, planning of the production, or a temporary lack of access (Clark & Wasow, 1998; Garret, 1982).

We are grateful to Sieb Nooteboom for making this suggestion.

However, see Lind et al.’s (2015) response regarding the detection rates.

We are grateful to an anonymous reviewer for making this suggestion.

However, another probable candidate for this role, which cannot be excluded at this time, is dopamine.

The matlab code for the simulation is available from osf.io/fraz9.
